# Synthesis, spectroscopic and crystallographic characterization of various cymantrenyl thio­ethers [Mn{C_5_H_*x*_Br_*y*_(SMe)*_z_*}(PPh_3_)(CO)_2_]

**DOI:** 10.1107/S205322962400603X

**Published:** 2024-07-05

**Authors:** Christian Klein-Hessling, Tobias Blockhaus, Karlheinz Sünkel

**Affiliations:** a Chemistry, Ludwig-Maximilians-University Munich, Butenandtstrasse 5-13, Munich, D-81377, Germany; Hong Kong University of Science and Technology, Hong Kong

**Keywords:** crystal structure, cymantrene, thio­ether, cyclopentadienyl, Hirshfeld surface

## Abstract

Starting from [Mn(C_5_H_4_Br)(PPh_3_)(CO)_2_] (**1a**), the cymantrenyl thio­ethers [Mn(C_5_H_4_SMe)(PPh_3_)(CO)_2_] (**1b**) and [Mn{C_5_H_4–*n*_Br(SMe)_*n*_}(PPh_3_)(CO)_2_] (*n* = 1 for com­pound **2**, *n* = 2 for **3** and *n* = 3 for **4**) were obtained, using either *n*-butyllithium (*n*-BuLi), lithium diiso­propyl­amide (LDA) or lithium tetra­methyl­piperidide (LiTMP) as base, followed by electrophilic quenching with MeSSMe. Stepwise consecutive reaction of [Mn(C_5_Br_5_)(PPh_3_)(CO)_2_] with *n*-BuLi and MeSSMe led finally to [Mn{C_5_(SMe)_5_}(PPh_3_)(CO)_2_] (**11**), only the fifth com­plex to be reported containing a perthiol­ated cyclo­penta­dienyl ring. The mol­ecular and crystal structures of **1b**, **3**, **4** and **11** were determined and were studied for the occurrence of S⋯S and S⋯Br inter­actions. It turned out that although some inter­actions of this type occurred, they were of minor importance for the arrangement of the mol­ecules in the crystal.

## Abstract 2

Several manganese com­plexes of the type [MnCp(PPh_3_)(CO)_2_] containing a cyclo­penta­dienyl (Cp) ligand with one to five SMe substituents were prepared and studied by X-ray crystallography. In these structures, a few S⋯S and/or S⋯Br inter­actions occur, and these are sometimes of significant importance for the arrangement of the mol­ecules in the crystal.

## Introduction

Aromatic thio­ethers, a long-known substance class, have attracted substanti­ally increased inter­est over the last 30 years. A quick search in *Scifinder* (accessed on February 15, 2024) showed that while the annual number of publications stayed around 25 until 1996, this number then started to increase exponentially and reached a maximum of 205 in 2019 and was still at 174 in 2023. The main reason for this development can be attributed to the vast number of applications aromatic thio­ethers have found in agricultural chemistry (Li *et al.*, 2021 ▸) and medicinal chemistry (Feng *et al.*, 2016 ▸), and their importance in natural product biosynthesis (Dunbar *et al.*, 2017 ▸). A special subgroup, bis­(ar­yl) thio­ethers, has also found increased inter­est due to their photochemical properties (Riebe *et al.*, 2017 ▸). While there are thousands of ‘purely organic’ aryl thio­ethers, the number of organometallic derivatives, particularly of the metallocene type, where a transition metal is π-coordinated to the aromatic part of the aryl thio­ether, is rather small. When it comes to persulfurated cyclo­penta­dienyl com­plexes, there are only four com­pounds known. Three contain the penta­kis­(methyl­sulfan­yl)cyclo­penta­dienyl ligand, [{C_5_(SMe)_5_}*ML_n_*] [*ML_n_* = Mn(CO)_3_ (Sünkel & Motz, 1988 ▸), RuCp* (Seneviratne & Winter, 1997 ▸) and FeCp (Blockhaus *et al.*, 2019 ▸)] and one contains the penta­kis­(phenyl­sulfan­yl)cyclo­penta­dienyl ligand, [{C_5_(SPh)_5_}FeCp] (Blockhaus *et al.*, 2019 ▸). Similarly, while there are *ca* 250 entries in *Scifinder* for the search mask ‘Cr(η^6^-C_6_R_5_S-C)’, there are only four entries for the benzene tris­(thio­ether) and none for any higher thiol­ated benzene derivatives. When looking for crystal structure determinations of π-coordinated cyclo­penta­dienyl thio­ethers in the Cambridge Structural Database (CSD; Groom *et al.*, 2016 ▸; accessed on February 15, 2024), one finds 301 entries for the search mask with one thio­ether function. Of these, 233 are ferrocene-, 14 ruthenocene and 13 cymantrene derivatives. We therefore decided to look at the viability of a synthesis of a penta­sulfurated cymantrene derivative where tri­phenyl­phosphane has substituted one carbonyl ligand and determine the crystal structures of these com­pounds.

**Figure 1:**
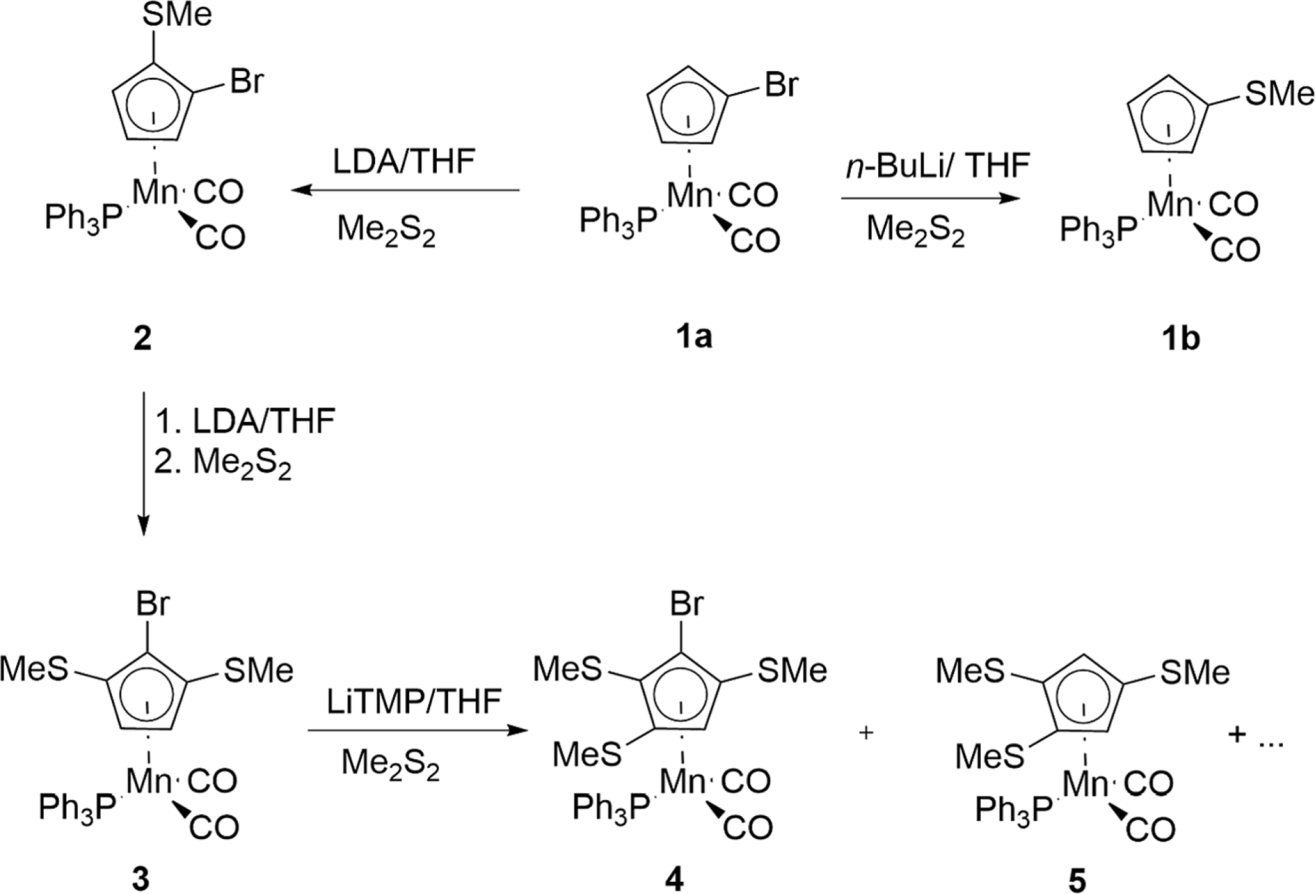
Li­thia­tion of [Mn(C_5_H_4_Br)(PPh_3_)(CO)_2_] (**1a**) followed by electrophilic quenching with Me_2_S_2_. Compounds **2** and **4** possess planar chirality and only one enanti­omer is shown.

The title com­pounds determined are dicarbon­yl[η^5^-1-(methyl­sulfan­yl)cyclo­penta­dien­yl](tri­phenyl­phosphane-κ*P*)manganese, [Mn(C_5_H_4_SMe)(PPh_3_)(CO)_2_] (**1b**), dicarbon­yl[η^5^-1-bromo-2-(methyl­sulfan­yl)cyclo­penta­dien­yl](tri­phenyl­phosphane-κ*P*)manganese cyclo­hexane 0.75-solvate [Mn{C_5_H_3_Br(SMe)}(PPh_3_)(CO)_2_],C_6_H_12_ (**2**), dicarbon­yl[η^5^-2-bromo-1,3-bis­(methyl­sulfan­yl)cyclo­penta­dien­yl](tri­phenyl­phosphane-κ*P*)manganese, [Mn{C_5_H_2_Br(SMe)_2_}(PPh_3_)(CO)_2_] (**3**), di­car­bon­yl[η^5^-2-bromo-1,3-bis­(methyl­sulfan­yl)cyclo­penta­dien­yl](tri­phenyl­phosphane-κ*P*)manganese, [Mn{C_5_H_2_Br(SMe)_2_}(PPh_3_)(CO)_2_] (**4**), and dicarbon­yl[η^5^-1,2,3,4,5-penta­kis­(methyl­sulfan­yl)cyclo­penta­dien­yl](tri­phenyl­phosphane-κ*P*)manganese, [Mn{C_5_(SMe)_5_}(PPh_3_)(CO)_2_] (**11**) (see Figs. 1 ▸ and 2 ▸)

## Experimental

### Synthesis and crystallization

The synthesis of com­pounds [Mn(C_5_H_4_Br)(PPh_3_)(CO)_2_] (**1a**) and [Mn(C_5_Br_5_)(PPh_3_)(CO)_2_] (**6**) was performed as described by us earlier (Klein-Hessling *et al.*, 2021 ▸). All reagents and solvents were commercially available and were used as received. Li­thia­tion reactions were performed under an N_2_ atmosphere, while the chromatographic purifications were performed in air.

#### Synthesis of [Mn(C_5_H_4_SMe)(PPh_3_)(CO)_2_], 1b

A solution of **1a** (0.050 g, 0.097 mmol) in tetra­hydro­furan (THF, 8 ml) was treated at 195 K with 2.5 *M n*-BuLi solution (0.040 ml, 0.10 mmol) with stirring for 30 min. MeSSMe (0.010 g, 0.10 mmol) was then added and the mixture was warmed gradually to room temperature within 16 h. The reaction mixture was filtered through a plug of silica gel and then evaporated *in vacuo*. The residue was taken up in the minimum amount of petroleum ether (PE) and placed on top of a silica-gel column. PE/CH_2_Cl_2_ (85:15 *v*/*v*) eluted a yellow band. Evaporation of the solvent *in vacuo* left **1b** as a yellow powder (yield: 0.040 g, 0.0820 mmol, 85%). For spectra, see Figs. S1–S3 and S27 in the supporting information.

^1^H NMR (CDCl_3_, 400 MHz): δ 7.52–7.31 (15H), 4.47 (2H), 4.04 (2H), 2.35 (3H). ^13^C{^1^H} NMR (CDCl_3_, 101 MHz): δ 232.2 (*d*, *J* = 25.9 Hz), 137.9 (*d*, *J* = 40.7 Hz), 133.0 (*d*, *J* = 9.9 Hz), 129.6, 128.2 (*d*, *J* = 8.9 Hz), 99.5, 83.6, 82.9 (*d*, *J* = 9.4 Hz), 19.0. ^31^P{^1^H} NMR (CDCl_3_, 162 MHz): δ 92.5. IR (ATR; cm^−1^): ν(CO) = 1927, 1862. MS (EI, 70 eV): *m*/*z* = 484.9 (*M*^+^), 428.1 (*M*^+^ – 2CO), 413.1 (*M*^+^ – 2CO – Me), 262.1 (PPh_3_), 183.0 (PPh_2_), 108.0 (PPh). Elemental analysis (EA) calculated (%) for C_26_H_22_MnO_2_PS: C 64.46, H 4.58, S 6.62; found: C 63.68, H 4.70, S 6.62.

#### Synthesis of [Mn{C_5_H_3_Br(SMe)}(PPh_3_)(CO)_2_], 2

A solution of **1a** (0.50 g, 0.97 mmol) in THF (15 ml) was treated at 195 K with 1.0 *M* lithium diiso­propyl­amide (LDA) solution (1.16 ml, 1.16 mmol) with stirring for 1 h. MeSSMe (0.10 ml, 1.25 mmol) was then added and the mixture was warmed gradually to room temperature within 16 h. The mixture was filtered through a plug of silica gel and then evaporated *in vacuo*. The residue was taken up in the minimum amount of PE and placed on top of a silica-gel column. PE/Et_2_O (85:15 *v*/*v*) eluted a yellow band. Evaporation of the solvent *in vacuo* left **2** as a yellow powder (yield: 0.43 g, 0.76 mmol, 79%). Recrystallization from PE yielded yellow crystals which were of insufficient quality for publication, due to severe disorder problems. For spectra, see Figs. S4–S6 in the supporting information.

^1^H NMR (CDCl_3_, 400 MHz): δ 7.53–7.29 (*m*, 15H), 4.46 (*s*, 1H), 4.36 (*s*, 1H), 3.69 (*s*, 1H), 2.36 (*s*, 3H). ^13^C{^1^H} NMR (CDCl_3_, 101 MHz): δ 231.4 (*d*, *J* = 23.6 Hz), 137.4 (*d*, *J* = 41.3 Hz), 133.1 (*d*, *J* = 10.5 Hz), 129.9, 128.4 (*d*, *J* = 9.5 Hz), 95.9, 89.6, 85.7, 84.9, 82.2, 19.9. ^31^P{^1^H} NMR (CDCl_3_, 162 MHz): δ 90.9. IR (ATR; cm^−1^): ν(CO) = 1935, 1874.

#### Synthesis of [Mn{C_5_H_2_Br(SMe)_2_}(PPh_3_)(CO)_2_], 3

A solution of **2** (0.43 g, 0.76 mmol) in THF (15 ml) was treated at 195 K with 1.0 *M* LDA solution (0.92 ml, 0.92 mmol) with stirring for 1 h. MeSSMe (0.080 ml, 1.00 mmol) was then added and the mixture was warmed gradually to room tem­per­ature within 16 h. The mixture was filtered through a plug of silica gel and then evaporated *in vacuo*. The residue was taken up in the minimum amount of PE and placed on top of a silica-gel column. PE/Et_2_O (85:15 *v*/*v*) eluted a yellow band. Evaporation of the solvent *in vacuo* left **3** as a yellow powder (yield: 0.29 g, 0.48 mmol, 63%). Recrystallization from PE yielded yellow crystals, which were suitable for X-ray dif­frac­tion and full structure refinement. For spectra, see Figs. S7–S9 in the supporting information.

^1^H NMR (CDCl_3_, 400 MHz): δ 7.52–7.45 (*m*, 6H), 7.39–7.35 (*m*, 9H), 3.93 (*s*, 2H), 2.23 (*s*, 6H). ^13^C{^1^H} NMR (CDCl_3_, 101 MHz): δ 230.8 (*d*, *J* = 23.4 Hz), 137.2 (*d*, *J* = 41.2 Hz), 133.2 (*d*, *J* = 10.7 Hz), 129.9, 128.3 (*d*, *J* = 9.5 Hz), 99.6, 89.5, 83.8, 18.9. ^31^P{^1^H} NMR (CDCl_3_, 162 MHz): δ 89.4. IR (ATR; cm^−1^): ν(CO) = 1941, 1880. MS (EI, 70 eV): *m*/*z* = 554.4 (*M*^+^ – 2CO), 539.4 (*M*^+^ – 2CO – CH_3_).

#### Synthesis of [Mn{C_5_HBr(SMe)_3_}(PPh_3_)(CO)_2_], 4

A solution of **3** (0.29 g, 0.48 mmol) in THF (10 ml) was treated at 195 K with a freshly prepared lithium tetra­methyl­piperidide (LiTMP) solution (1.19 mmol in 2 ml THF) with stirring for 1 h. MeSSMe (0.110 ml, 1.19 mmol) was then added and the mix­ture was warmed gradually to room temperature within 16 h. The mixture was filtered through a plug of silica gel and then evaporated *in vacuo*. NMR and mass spectra (see Figs. S10, S11 and S28) showed this product to be a mixture of at least four com­pounds. The MS showed only **3**, **4** and **5**, but of course without any indication of stereochemistry; the ^31^P NMR spectrum showed five signals of relevant intensity, assignable to compounds **2**, **3**, **4** and **X**, and one further unknown, possibly **5**. In the ^1^H NMR spectrum, there are several signals in the Cp region (5.0–3.7 ppm), that have apparently no counterpart in the SMe region of the spectrum. The residue was taken up in the minimum amount of PE and placed on top of a silica-gel column. PE/Et_2_O (85:15 *v*/*v*) eluted two yellow bands. The first (F1) gave apparently unreacted starting material **3** (yield: 0.060 g, 21%; Fig. S12). The second still yielded a mixture and was therefore re-chromatographed, using PE/Et_2_O (1:1 *v*/*v*) as eluent. The first fraction (F2.1) left, after full evaporation of the solvent, **4** as a yellow powder (yield: 0.10 g, 0.15 mmol, 31%; Figs. S13 and S14). The second fraction (F2.2) left, after evaporation of the solvent, a yellow product, which, according to its NMR spectra (Figs. S15 and S16), was still a mixture of two main products, **4** and ‘**X**’, together with small amounts of unidentified by-products. Although com­pound **X** could not be isolated in a pure form, the appearance of its ^1^H NMR spectrum suggests that it is a stereoisomer of **4**, like [{C_5_H(Br-1)[(SMe)_3_-2,3,4]}Mn(PPh_3_)(CO)_2_].

For **4**, ^1^H NMR (CDCl_3_, 400 MHz): δ 7.59–7.46 (*m*, 6H), 7.43–7.32 (*m*, 9H), 3.82 (*s*, 1H), 2.39 (*s*, 3H), 2.05 (*s*, 3H), 1.97 (*s*, 3H). ^31^P{^1^H} NMR (CDCl_3_, 162 MHz): δ 87.0. IR (ATR; cm^−1^): ν(CO) = 1941, 1885.

For **X**, ^1^H NMR (CDCl_3_, 400 MHz): δ 4.33 (*s*, 1H), 2.32/2.24/2.14 (3*s*, 3 × 3H). ^31^P{^1^H} NMR (CDCl_3_, 162 MHz): δ 88.6.

#### One-pot reaction of [Mn(C_5_Br_5_)(PPh_3_)(CO)_2_] (6) with excessive *n*-butyl­lithium and MeSSMe: synthesis of [Mn{C_5_Br_3_(SMe)_2_}(PPh_3_)(CO)_2_], 7, and [Mn{C_5_(SMe)_5_}(PPh_3_)(CO)_2_] (11)

Method (*a*). A solution of **6** (0.20 g, 0.24 mmol) in THF (10 ml) was treated at 195 K with 2.5 *M n*-BuLi solution (0.20 ml, 0.50 mmol) with stirring for 60 min. MeSSMe (0.040 ml, 0.50 mmol) was then added and the mixture was warmed gradually to room temperature within 16 h. The mixture was filtered through a plug of silica gel and then evaporated *in vacuo*, yielding 0.12 g of product. NMR (Figs. S17 and S18) and MS (Fig. S29) spectra showed this product to be a mixture of at least four com­pounds, **7**–**10**, with com­pound **7** as the dominant product. The residue was taken up in the minimum amount of PE and placed on top of a silica-gel column. PE/Et_2_O (90:10 *v*/*v*) eluted a yellow band. After evaporation of the solvent *in vacuo*, **7** (still impure) was left as a yellow powder (yield: 0.16 g, <0.21 mmol, <87%). Part of this product (0.060 g, <0.08 mmol) was dissolved in THF (10 ml) and treated at 183 K with BuLi solution (0.030 ml, 0.075 mmol) with stirring for 30 min. MeSSMe (0.010 ml, 0.12 mmol) was then added and the mixture was warmed to room temperature within 16 h. The mixture was filtered through a plug of silica gel. Evaporation of the solvent left a yellow powder (0.020 g). This crude product was redissolved in THF (8 ml) and treated at 183 K with BuLi solution (0.010 ml, 0.025 mmol) with stirring for 60 min. Then, still at 183 K, MeSSMe (0.003 ml, 0.04 mmol) was added and the temperature was raised to ambient temperature within 16 h. After com­plete evaporation of the solvent, the residue was taken up in the minimum amount of PE and placed on top of a silica-gel chromatography column. Elution with PE/Et_2_O (9:1 *v*/*v*) produced two fractions. Evaporation of the second fraction (F2) left a yellow powder (0.010 g). NMR spectroscopy (see Figs. S22 and S23) showed this product to be nearly pure **11** contaminated with an unknown product ‘**Y**’. Although the latter could not be isolated in a pure form, its ^1^H NMR data suggest its formulation as [Mn{C_5_H(SMe)_4_}(PPh_3_)(CO)_2_].

Method (*b*). The conditions of method (*a*) were slightly changed, using 0.19 ml *n*-BuLi solution and stirring for only 30 min. The NMR spectra of the crude product showed the presence of only two com­pounds, **7** and **8** (Figs. S19–S21). The residue was redissolved in THF (10 ml) and treated at 183 K with 2.5 *M n*-BuLi solution (0.19 ml, 0.48 mmol) with stirring for 30 min. MeSSMe (0.050 ml, 0.60 mmol) was then added at this temperature. The mixture was warmed gradually to room temperature within 16 h with continuous stirring and was then filtered through a plug of silica gel and evaporated *in vacuo*. The residue was taken up in the minimum amount of PE and placed on top of a silica-gel column. PE/Et_2_O (90:10 *v*/*v*) eluted a yellow band. Evaporation of the solvent *in vacuo* left **11** as a yellow powder (yield: 0.050 g, 0.075 mmol, 31%). Recrystallization from PE gave yellow crystals suitable for X-ray diffraction. For spectra, see Figs. S24–S26 and S30.

For **7**, ^1^H NMR (CDCl_3_, 400 MHz): δ 7.54–7.33 (*m*, 15H), 2.26 (*s*, 6H). ^13^C{^1^H} NMR (CDCl_3_, 101 MHz): δ 229.7 (*d*, *J* = 24.4 Hz), 134.5 (*d*, *J* = 42.4 Hz), 133.9 (*d*, *J* = 10.6 Hz), 130.1, 128.3 (*d*, *J* = 9.7 Hz), 99.4, 94.2, 90.7, 19.5. ^31^P{^1^H} NMR (CDCl_3_, 162 MHz): δ 82.2. IR (ATR; cm^−1^): ν(CO) = 1942, 1889. MS (EI, 70 eV): *m*/*z* = 767.9 (*M*^+^), 711.8 (*M*^+^ – 2CO), 696.8 (*M*^+^ – 2CO – Me), 677.9 (*M*^+^ – 2CO – 2Me), 262.0 (PPh_3_), 183.0 (PPh_2_), 108.0 (PPh). HRMS (EI): calculated for C_27_H_21_O_2_PS_2_Mn^79^Br_3_: *m*/*z* = 763.7651; found: 763.7654.

For **8**, ^1^H NMR (CDCl_3_, 270 MHz): δ 7.55–7.16, 4.10 (*s*, 1H), 2.35 (*s*, 3H). ^31^P{^1^H} NMR (CDCl_3_, 109 MHz): δ 85.8. MS (EI, 70 eV): *m*/*z* = 663.8/665.7 (*M*^+^ – 2CO), 648.7/650.8 (*M*^+^ – 2CO – Me).

For **9**, ^1^H NMR (CDCl_3_, 270 MHz): δ 7.55–7.16, 2.36, 2.30. ^31^P{^1^H} NMR (CDCl_3_, 109 MHz): δ 81.9. MS (EI, 70 eV): *m*/*z* = 677.8 (*M*^+^ – 2CO), 662.8 (*M*^+^ – 2CO – Me), 647.8 (*M*^+^ – 2CO – 2Me), 416.0 (*M*^+^ – 2CO – PPh_3_), 401.0 (*M*^+^ – 2CO – PPh_3_ – Me).

For **10**, MS (EI, 70 eV): *m*/*z* = 631.8 (*M*^+^ – 2CO), 616.8 (*M*^+^ – 2CO – Me), 601.8 (*M*^+^ – 2CO – 2Me), 370.0 (*M*^+^ – 2CO – PPh_3_).

For **11**, ^1^H NMR (CDCl_3_, 400 MHz): δ 7.55–7.48 (*m*, 6H), 7.40–7.34 (*m*, 9H), 2.38 (*s*, 15H). ^13^C{^1^H} NMR (CDCl_3_, 101 MHz): δ 135.9 (*d*, *J* = 41.4 Hz), 133.9 (*d*, *J* = 10.4 Hz), 129.9, 128.1 (*d*, *J* = 9.6 Hz), 102.7, 20.2. ^31^P{^1^H} NMR (CDCl_3_, 162 MHz): δ 82.3. IR (ATR; cm^−1^): ν(CO) = 1939, 1885. MS (EI, 70 eV): *m*/*z* = 668.1 (*M*^+^), 612.1 (*M*^+^ – 2CO), 597.0 (*M*^+^ – 2CO – Me), 582.0 (*M*^+^ – 2CO – 2Me), 262.0 (PPh_3_), 183.0 (PPh_2_), 108.0 (PPh).

For **Y**, ^1^H NMR (CDCl_3_, 400 MHz): δ 7.55–7.48 (*m*, 6H), 7.40–7.34 (*m*, 9H), 5.49 (*s*, 1H), 2.44 (*s*, 6H), ≃ 2.38 (hidden under signal of **11**). ^31^P{^1^H} NMR (CDCl_3_, 162 MHz): δ 87.3.

### Refinement

Crystal data, data collection and structure refinement details are summarized in Table 1 ▸. All structures were solved with *SHELXT* (Sheldrick, 2015*a* ▸) and refined with *SHELXL* (Sheldrick, 2015*b* ▸). In the refinement of all structures, several low-angle reflections had to be omitted due to beam-stop inter­ferences (five in **1b**, five in **3**, six in **4** and five in **11**). For com­pound **4**, the *SHELXT* solution suggested one Mn, one Br and four S atoms. One of the S atoms turned out to actually be a P atom. Another S atom had significantly higher electron density than the remaining two. It was concluded that this was due to positional disorder with a Br atom. A first difference Fourier synthesis, which showed a residual electron-density peak at a distance of 1.800 Å from the ‘bromine’ atom, con­firmed this assumption. No significant electron density was found near the Cp-ring ‘CH carbon’. Therefore, a model was re­fined where the two ‘inner’ ring substituent atoms were both in part S and in part Br atoms. Refinement gave a 76:24 disorder in favour of the ‘original’ structure solution. In order to stabilize the refinement, some SADI restraints had to be em­ployed (SADI restrains particular distances to be identical within certain standard deviations).

The data for com­pound **2** are included for com­parison. While there was no problem obtaining the solution, refinement proceeded only with difficulty. First, three medium-intensity peaks close to an inversion centre turned up in a difference Fourier synthesis, which when connected had the appearance of a cyclo­hexane ring. Although cyclohexane was not explicitly used during the synthesis, it may have been part of the petroleum ether that was used for recrystallization. Ap­parently, ‘petroleum ether’ is not a particular compound, but a mixture of hydrocarbons within a specific range of boiling points. Therefore, the three atoms were refined iso­tropi­cally with a common displacement parameter, and the site occupancy factor (s.o.f.) was refined as well. Refinement gave a value for the s.o.f. of *ca* 0.75, with a reasonable displacement parameter. Then the s.o.f. was fixed at 0.75 and the displacement parameters were allowed to re­fine freely, first isotropically and then anisotropically. Finally, methyl­ene H atoms were added according to the standard riding model of *SHELXL*. While the s.o.f. and displacement parameters ap­pear reasonable, some of the bond lengths appear unreasonably short. One possible explanation might be conformational disorder within the cyclohexane ring, which is quite usual for this molecule. The quality of the data set, however, did not allow for a proper resolution of this disorder. At the same time as the appearance of the solvent mol­ecule, a medium-intensity peak was also localized close to atom H3 of the cyclo­penta­dienyl ligand, with a distance of approximately 1.85 Å to atom C3. As com­pound **2** possesses planar chirality, we assumed this electron density derived from an alternative bromine position, corresponding to a rotational isomer of the enanti­omer of the major orientation (of course, in a centrosymmetric space group, both enanti­omers are present anyway). Again, both bromine positions were given a common displacement parameter, and their s.o.f. values were refined. This resulted in an s.o.f. value of only 0.045 for the minor orientation. Then the s.o.f. values were fixed at 0.955 and 0.045, respectively, and the displacement parameters were refined, first isotropically and then anisotropically. The corresponding H atoms were positioned according to the riding model. After this ‘problem’ was solved, another one appeared. A rather large residual electron-density peak turned up only 0.82 Å from the S atom, 1.73 Å from atom C2 and 0.71 Å on the distal side of the Cp ring. We could not find any explanation for this observation. The distance from sulfur is too small to be a methyl group or O atom, and the distance from the Cp plane is too large for a ring substituent. We also tried a refinement without the cyclohexane, using the SQUEEZE (Spek, 2015 ▸) model available in *PLATON* (Spek, 2020 ▸). However, this refinement neither provided better statistics nor change anything about the residual high electron density next to the S atom. With this unexplained residual electron density in mind, we refrained from any further structure discussion. However, displacement ellipsoid plots of the current ‘best’ solution are displayed in the supporting information.

## Results and discussion

### Synthesis

**Figure 2:**
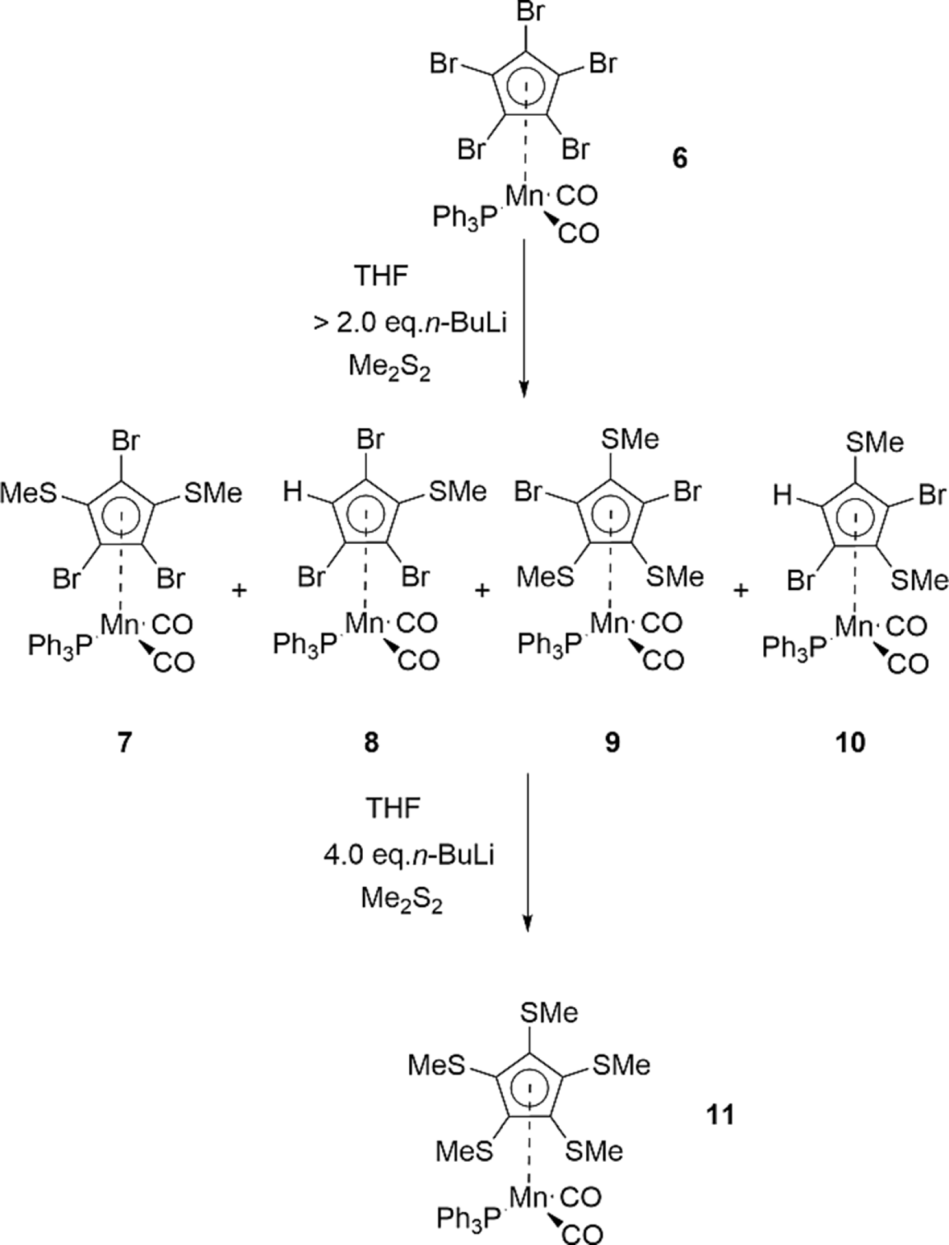
One-pot li­thia­tion of [Mn(C_5_Br_5_)(PPh_3_)(CO)_2_] (**6**) followed by electrophilic quenching with Me_2_S_2_. Compounds **8** and **10** possess planar chirality and only one enanti­omer is shown.

There are numerous synthetic pathways towards aryl thio­ethers. Some recent approaches have been the catalyzed or uncatalyzed methyl­thiol­ation of aryl iodides or bromides (Wang *et al.*, 2020 ▸), chlorides (Schmiedtchen *et al.*, 2023 ▸) or fluorides (Mörsel *et al.*, 2023 ▸), or the visible-light-mediated alkyl­ation of thio­phenols (Cai *et al.*, 2021 ▸). However, the reaction conditions used in these procedures are unfortunately not applicable neither for the ‘free’ substituted cyclo­penta­dienide nor in the system [MnCp(PPh_3_)(CO)_2_], due to rapid decom­position. Therefore, we chose two approaches that had been successful in the ferrocene (Blockhaus *et al.*, 2019 ▸) and the [MnCp(CO)_3_] systems (Sünkel & Motz, 1988 ▸). For the first approach, we used [Mn(C_5_H_4_Br)(PPh_3_)(CO)_2_] as the starting material (Fig. 1 ▸), while for the second, [Mn(C_5_Br_5_)(PPh_3_)(CO)_2_] was employed (Fig. 2 ▸). It should be mentioned that the photolysis of [Mn{C_5_(SMe)_5_}(CO)_3_] in the presence of PPh_3_ (like in the first synthesis of **1b**; Kursanov *et al.*, 1970 ▸) might work as well, but we didn’t try this.

Treatment of **1a** with *n*-BuLi in THF followed by addition of Me_2_S_2_ led to the product of Br–Li exchange, *i.e.* com­pound **1b**, in 85% yield. This com­pound has been known for over 50 years and had originally been prepared by photolysis of [Mn(C_5_H_4_SMe)(CO)_3_] in the presence of PPh_3_ (Kursanov *et al.*, 1970 ▸). When LDA was used as the base instead of *n*-BuLi, **1a** was deprotonated in the α-position and, after electrophilic quenching with Me_2_S_2_, the disubstituted com­plex **2** was obtained in 79% yield, as an enanti­omeric pair due to the planar chirality. Renewed treatment with LDA and Me_2_S_2_ gave the tris­ubstituted com­pound **3** in 63% yield, apparently exclusively as the 1-bromo-2,5-bis­(methyl­sulfan­yl)- isomer. When **3** was treated with LDA/Me_2_S_2_, apparently only unreacted **3** could be recovered. Therefore, we decided to use the stronger base LiTMP, in 2.5 equivalents before adding Me_2_S_2_. NMR and mass spectrometric examination of the crude reaction product showed the presence of com­pound **4**, together with unreacted starting material **3**, presumably **5** and other unknown com­pounds. Chromatography allowed the isolation of rather pure com­pound **4**, albeit in rather low yield (31%). The parallel formation of **5** hints at the occurrence of ‘halogen dance’ reactions (Blockhaus *et al.*, 2020 ▸).

We then turned to an alternative approach, similar to that described by us for the synthesis of [Mn{C_5_(SMe)_5_}(CO)_3_]. We assumed that treatment of [Mn(C_5_Br_5_)(PPh_3_)(CO)_2_] (**6**) with two equivalents of *n*-BuLi/MeSSMe would yield exclusively the 1,3-disubstituted com­plex **7** (Fig. 2 ▸). However, it turned out that the reaction was extremely sensitive to the relative stoichiometry of the reactants, the reaction time and the presence of moisture. While with apparently exact stoichiometry, a mixture of com­pounds **7** and **8** was obtained, only a slight excess of *n*-BuLi gave a mixture of at least five com­pounds, of which **7**–**10** could be identified by mass spectrometry. It was not possible to isolate any of these com­pounds in sufficient purity for elemental analysis. However, characterization by ^1^H NMR and ^31^P NMR spectroscopy, as well as mass spectrometry, was possible. The unexpected formation of com­pounds **8**–**10** is most likely due to a combination of hydrolysis and ‘halogen dance’ or even ‘sulfur dance’ reactions (Gahlot *et al.*, 2024 ▸). Therefore, we decided to use these mixtures for further treatment with an excess of *n*-BuLi/MeSSMe, which yielded rather pure com­pound **11** (Fig. 2 ▸). Further purification steps *via* chromatography and recrystallization gave this com­pound as a monocrystalline material that could be studied by X-ray diffraction.

### Mol­ecular structures

#### [Mn(C_5_H_4_SMe)(PPh_3_)(CO)_2_], 1b

**Figure 3:**
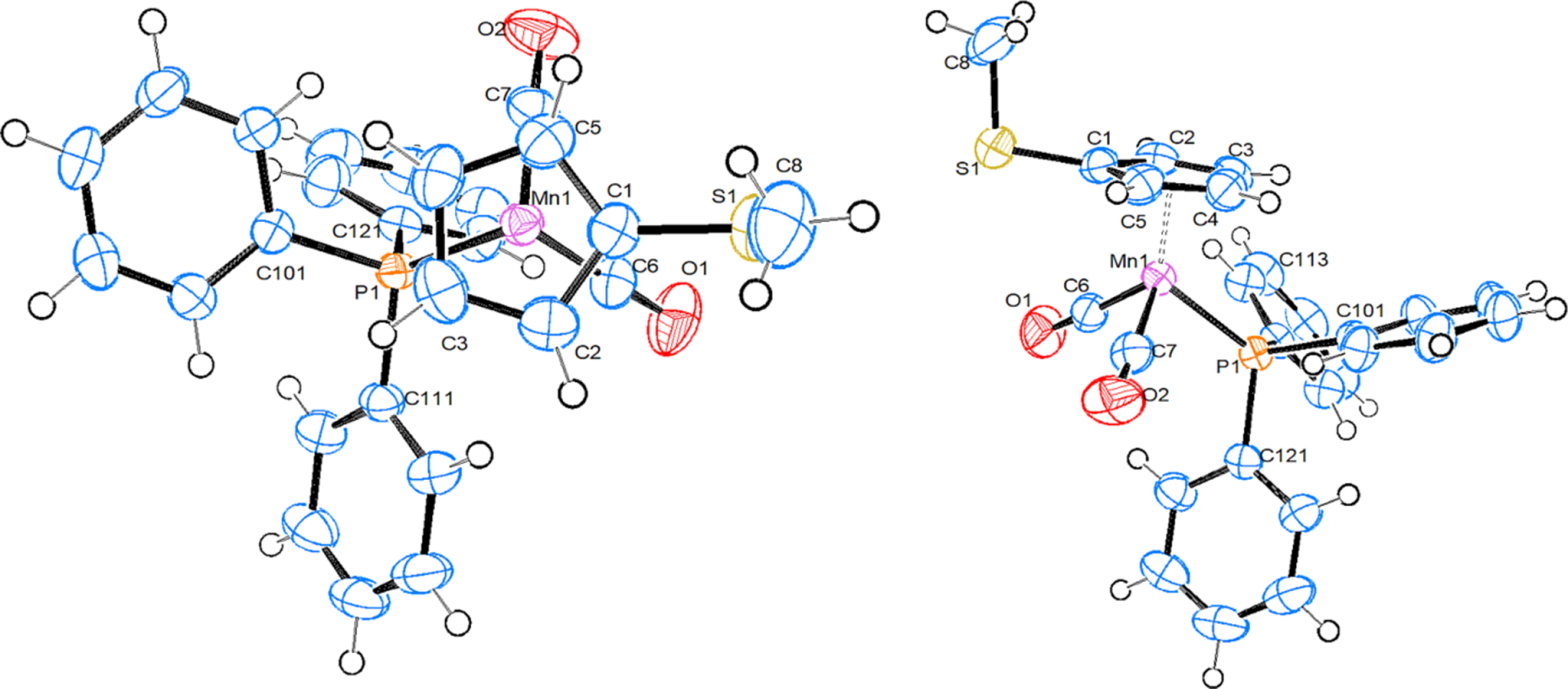
Top and side views of the mol­ecular structure of **1b**, with displacement ellipsoids drawn at the 50% probability level.

Compound **1b** crystallizes in the monoclinic space group *P*2_1_/*c*, with one mol­ecule in the asymmetric unit (Fig. 3 ▸). The bond parameters of **1b**, together with those of the other structures described here, can be found in Table 2 ▸. There are no unusual features. In com­parison with the ring-unsubstituted parent com­pound (Sünkel & Klein-Hessling, 2021 ▸), the Mn—CO and Mn—Ct_Cp_ (Ct_Cp_ is the centroid of the cyclo­penta­dienyl ring) distances are nearly identical, and the Mn—P bond is slightly elongated. In both com­pounds, one Mn—CO bond bis­ects one cyclo­penta­dienyl C—C bond, while the other Mn—CO and the Mn—P bond nearly eclipse a C—H bond of the ring. The SMe group in **1b** is in a relative *trans* position with respect to the PPh_3_ ligand. The methyl group at sulfur is in an axial position. For com­parison, the only two mononuclear structures containing a C_5_H_4_SMe ligand in the CSD, *i.e.* [Os(C_5_H_4_SMe)(CO)(PPh_3_)_2_]^+^ (CSD refcode ILORUX) and [Os(C_5_H_4_SMe)(CO)(PPh_3_)I] (ILOSAE), contain an equatorial and an axial methyl group, respectively (Johns *et al.*, 2010 ▸).

#### [Mn{C_5_H_2_Br(SMe)_2_}(PPh_3_)(CO)_2_], 3

**Figure 4:**
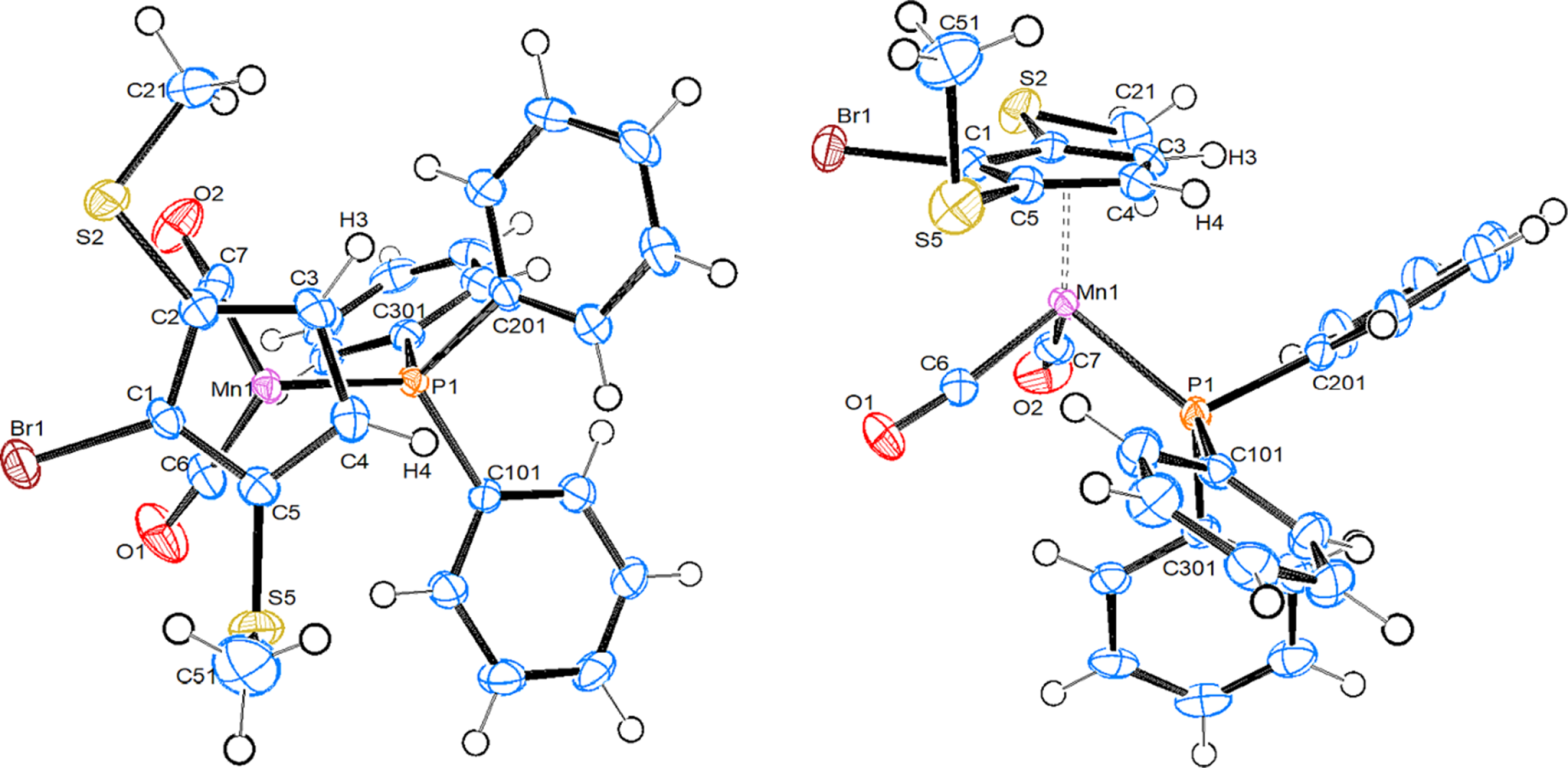
Top and side views of the mol­ecular structure of com­pound **3**, with displacement ellipsoids drawn at the 50% probability level.

Compound **3** crystallizes in the ortho­rhom­bic space group *Pbca*, with one mol­ecule in the asymmetric unit (Fig. 4 ▸). The bond parameters can be found in Table 2 ▸. Again, there are no unusual features. This time, the C—Br bond is in a relative *trans* position with respect to the Mn—P bond. One methyl group is in an axial position at atom S5, while the other at S2 is in an equatorial position. The Mn—P, Mn—CO and Mn—Ct_Cp_ bonds are virtually identical with the corresponding bond lengths in **1b**. The same is true for the C_Cp_—S and S—CH_3_ bonds. The bond angle at the S atom with the equatorial methyl group is significantly larger than the corresponding angle with the axial methyl group, which in turn is identical to the corresponding angle in com­pound **1b**.

#### Mn{C_5_HBr(SMe)_3_}(PPh_3_)(CO)_2_], 4

**Figure 5:**
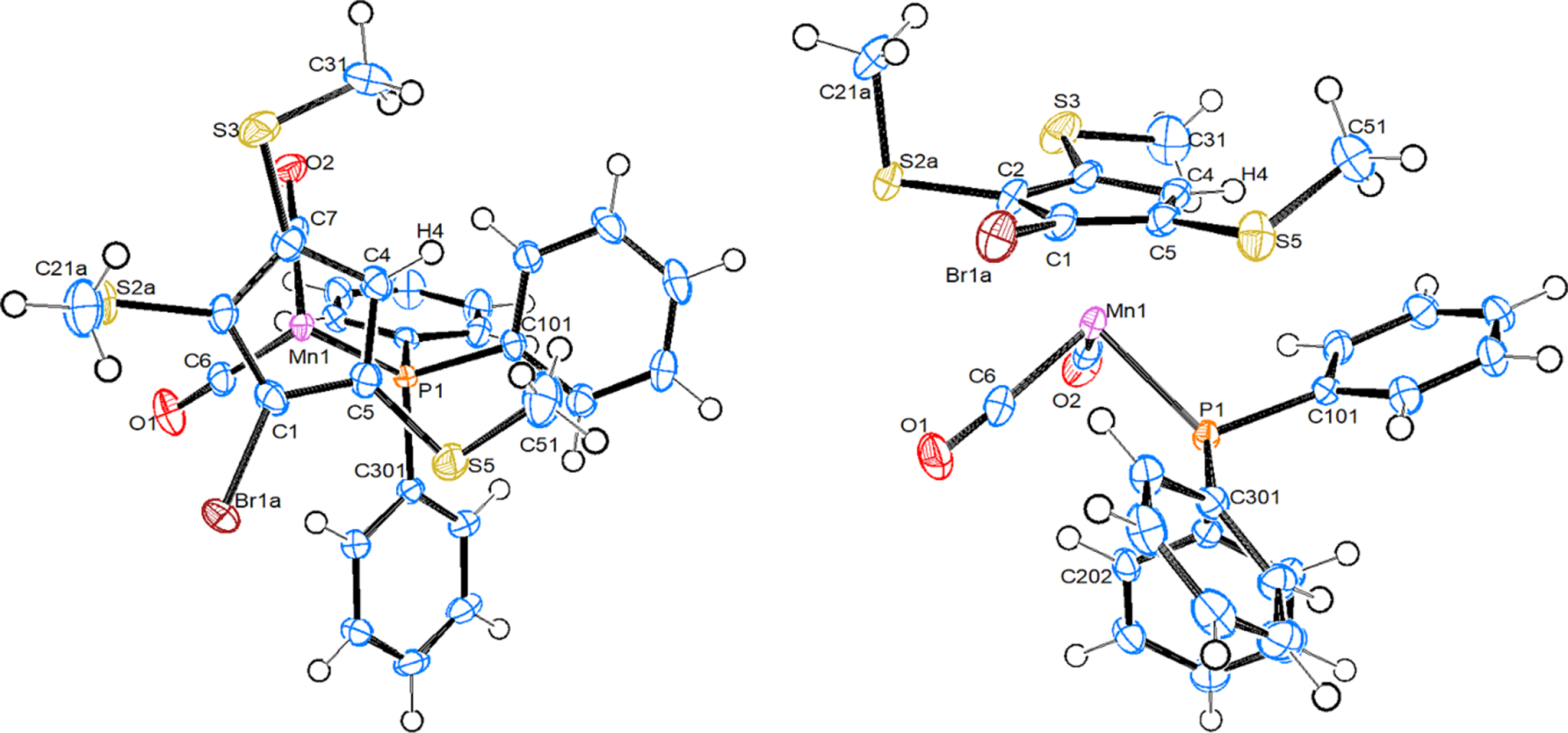
Top and side views of the mol­ecular structure (major com­ponent) of com­pound **4**, with displacement ellipsoids drawn at the 50% probability level. Only one enanti­omer of this planar chiral com­pound is shown.

Compound **4** crystallizes in the monoclinic space group *P*2_1_/*n*, with one mol­ecule in the asymmetric unit (Fig. 5 ▸). There is a 76:24 disorder (*i.e.* approximately 3:1) between the Br atom and the vicinal ‘inner’ methyl­sulfanyl group. Since com­pound **4** is planar chiral, this disorder resembles an unsymmetrical dis­order between the two enanti­omers.

In com­parison with the structures of **1b** and **3**, the Mn—P, Mn—CO and Mn—Ct_Cp_ bonds are slightly (but significantly, Δ > 5σ) elongated (Table 2 ▸). The two ‘outer’ methyl­sulfanyl groups are equatorial, with both methyl groups directed towards the unsubstituted C—H bond, while the ‘inner’ methyl group is in an axial position, directed away from the Mn atom. A little bit surprising is the observation that the Mn—P bond nearly eclipses one C_Cp_—S bond, instead of the neighbouring C—H bond, as one might expect. Thus, a rather short S⋯P distance of 3.601 (1) Å results, which is identical to the sum of the van der Waals radii. In addition, all S atoms are significantly closer to the Mn atom than the sum of their van der Waals radii (3.80 Å). This feature is more distinct for the S atoms with axial methyl groups (*R* ≃ 3.47 Å) than for those with equatorial methyl groups (*R* ≃ 3.57 Å), and is also observed in the structures of **1b** and **3**.

#### [Mn{C_5_(SMe)_5_}(PPh_3_)(CO)_2_], 11

**Figure 6:**
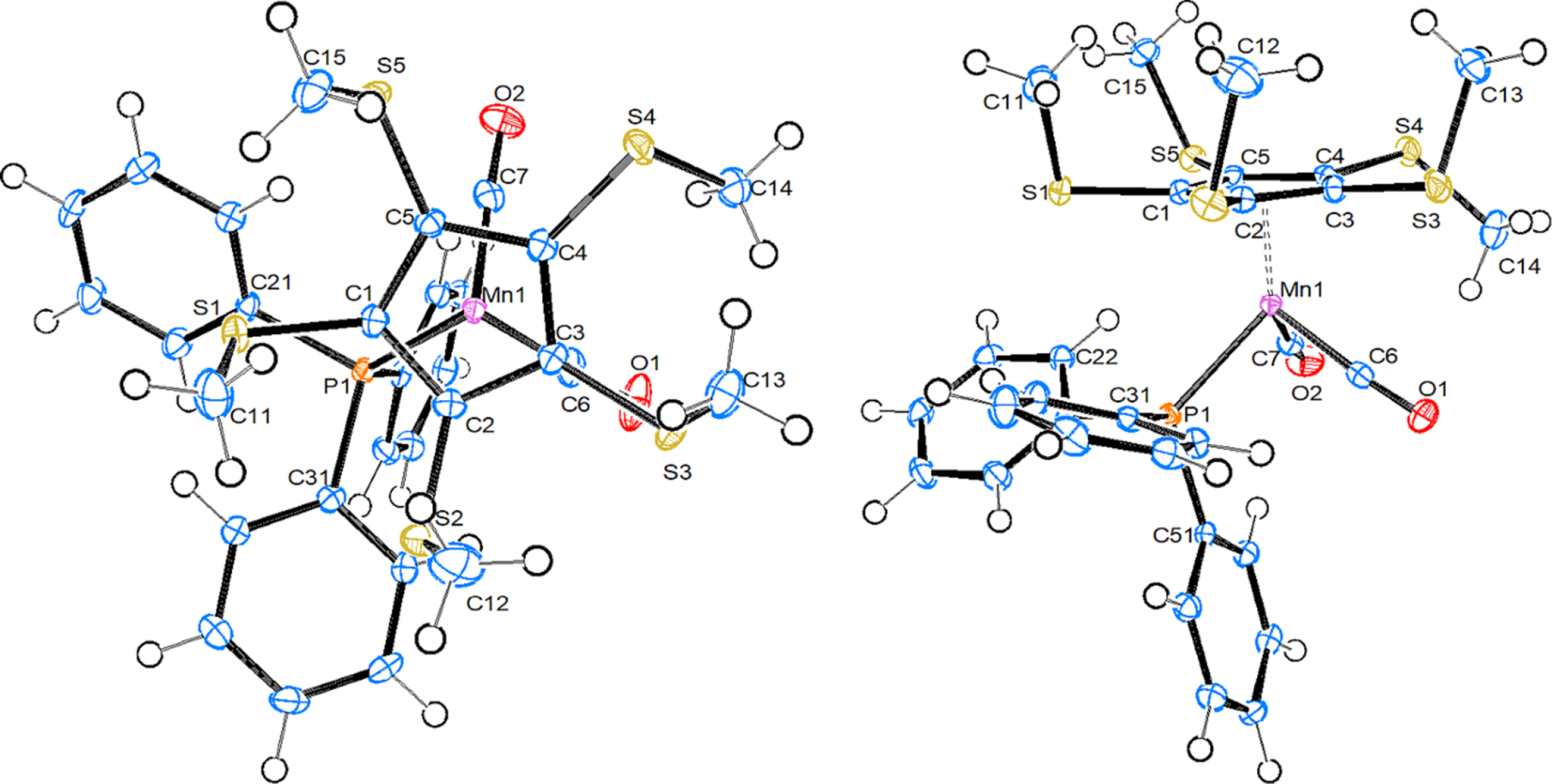
Top and side views of the mol­ecular structure of com­pound **11**, with displacement ellipsoids drawn at the 50% probability level.

Compound **11** crystallizes in the monoclinic space group *P*2_1_/*c*, with one mol­ecule in the asymmetric unit (Fig. 6 ▸). The Mn—P bond length in **11** is longer than in the other three com­pounds, while the Mn—CO and Mn—Ct_Cp_ distances are very similar to the values found in **4**. All methyl­sulfanyl groups are significantly tilted away from an ‘ideal’ axial position (C—C_Cp_—S—C_Me_ in the range between 50 and 68° *versus* 92° in **1b**). Four methyl groups are directed away towards the distal side of the cyclo­penta­dienyl ring, while one is on the proximal side. This can be com­pared to the structures of [Mn{C_5_(SMe)_5_}(CO)_3_], where there are three distal and two proximal SMe groups (Sünkel & Motz, 1988 ▸), and of [Fe{C_5_(SMe)_5_}(C_5_H_5_)], where all the SMe groups are in distal positions (Blockhaus *et al.*, 2019 ▸). This orientation with four methyl groups on one side of the ring and one methyl group on the other resembles, however, the situation found in the structure of the uncom­plexed ‘free’ anion (Wudl *et al.*, 1981 ▸). Theoretical studies of the conformational preferences of poly(methyl­sulfan­yl)ben­zenes, including hexa­kis­(methyl­sulfan­yl)benzene, have been reported (Lumbroso *et al.*, 1986 ▸; Fleurat-Lessard & Volatron, 2009 ▸), but to our knowledge no such studies of poly­thiol­ated cyclo­penta­ienyl rings exist. All the Mn⋯S distances are in the range 3.52–3.59 Å and are thus significantly shorter than the sum of their van der Waals radii (3.80 Å). For com­parison, in [Mn{C_5_(SMe)_5_}(CO)_3_], the Mn⋯S distances range from 3.39 to 3.59 Å. Still, it seems unlikely that there is explicit bonding between Mn and S, as such distances are also the simple geometrical result of π-bonding of the substituted Cp ring to the metal.

### Inter­molecular contacts and Hirshfeld analysis

**Figure 7:**
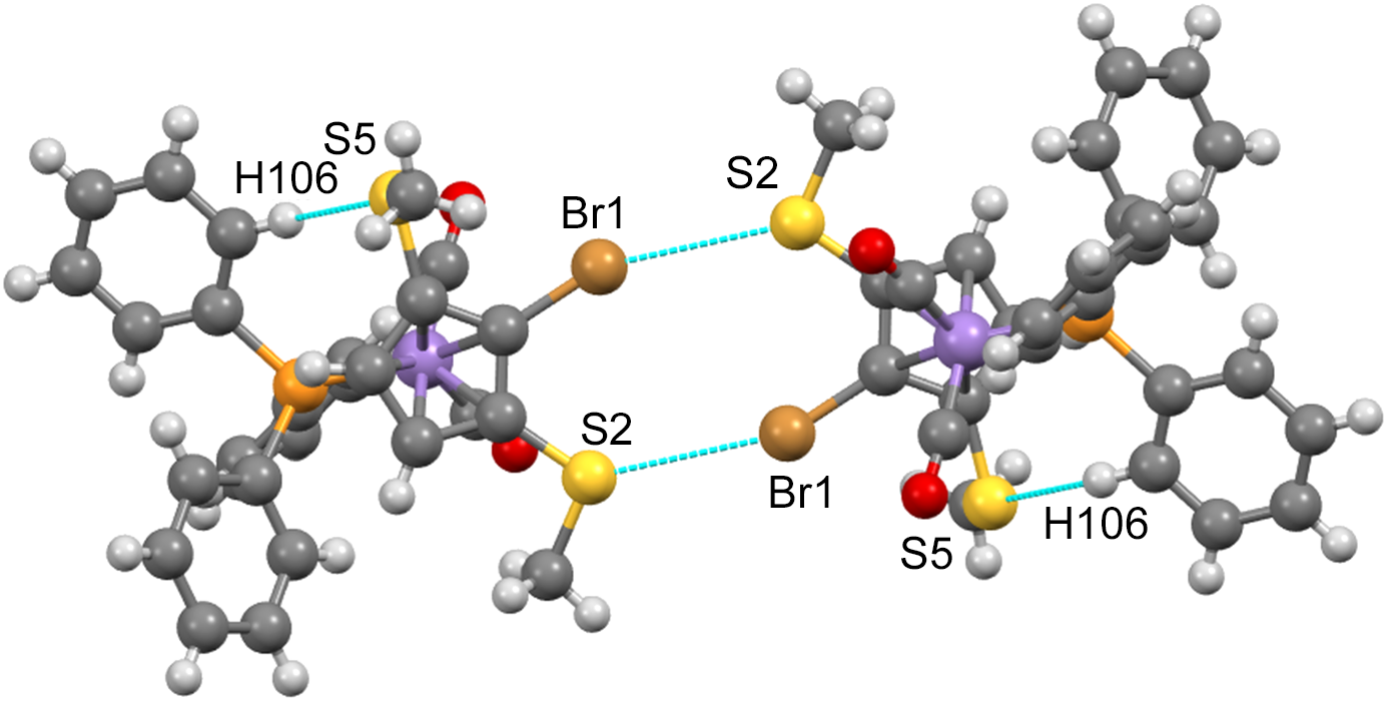
Dimer formation in the crystal of **3**,

The importance of inter­molecular contacts, also termed ‘noncovalent inter­actions’, for the build-up of crystal structures is undisputed. While the near omnipresence of hydrogen bonds has been known for a long time (mainly due to their structure-directing effects in biomolecules), in recent decades it was recognized that inter­actions involving halogens, chalcogens and even pnictogens and tetrel elements also have a great influence on the mutual arrangements of mol­ecules in crystals and the terms ‘halogen bond’, ‘chalcogen bond’, ‘pnictogen bond’ and ‘tetrel bond’ were created (Brammer *et al.*, 2023 ▸; Vogel *et al.*, 2019 ▸; Scheiner, 2023 ▸; Scilabra *et al.*, 2019 ▸; Mahmudov *et al.*, 2022 ▸). For the present study, we looked first only at the inter­actions involving S and/or Br atoms, using the corresponding feature in *Mercury* (Macrae *et al.*, 2020 ▸). In com­pound **1b**, no such inter­actions are observed. However, in com­pound **3**, double S⋯Br contacts of 3.414 Å, well below the sum of the van der Waals radii, lead to the formation of ‘dimers’ (Fig. 7 ▸).

The C—Br—S angle at Br1 is 153.6 (1)°, while the C—S—Br angles at S2 are 128.0 (1) and 128.8 (1)°. Atom S5 is not involved in such inter­actions; however, it accepts a hydrogen bond from a phenyl C—H group.

Compound **4** was not included for this study, due to the presence of the S/Br disorder, which did not allow a proper resolution of the relative contributions of these elements.

**Figure 8:**
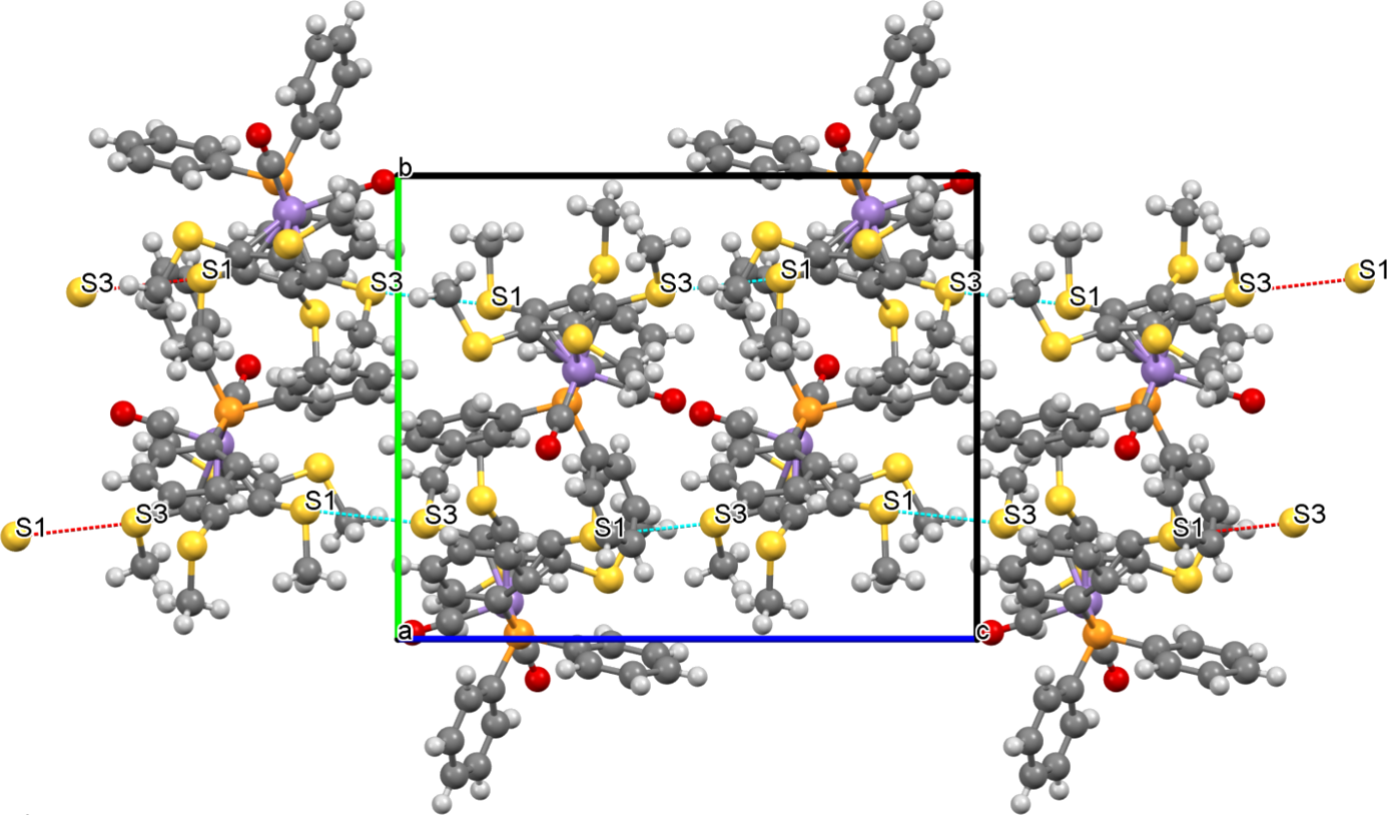
Packing plot of **11**, viewed along the *a* axis, showing the inter­molecular S⋯S contacts.

Compound **11** displays a mol­ecular chain in the crystallographic *c* direction, which is held together *via* weak S⋯S contacts (distance of 3.588 Å between atoms S1 and S3, just below the sum of the van der Waals radii). The C_Cp_—S—S angles at S1 and S3 are 139.8 (1) and 168.4 (1)°, respectively. Atoms S2 and S5 serve as hydrogen-bond acceptors towards two arene C—H bonds (Fig. 8 ▸). For com­parison, in closely related [Mn{C_5_(SMe)_5_}(CO)_3_] (Sünkel & Motz, 1988 ▸), only dimer formation *via* an S⋯S inter­action between inversion-related S atoms (3.510 Å) is observed. On the other hand, penta­kis­(methyl­sulfan­yl)ferrocene employs four S atoms for the formation of parallel undulating (wavy) chains along *b* using double S⋯S bridges on both sides (Blockhaus *et al.*, 2019 ▸).

**Figure 9:**
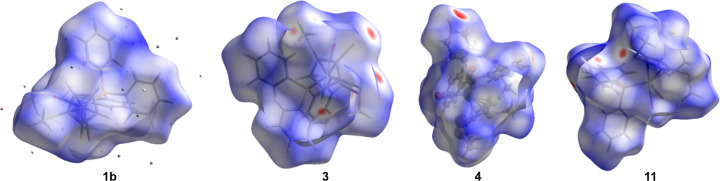
The Hirshfeld surfaces of **1b**, **3**, **4** and **11** (from left to right).

**Figure 10:**
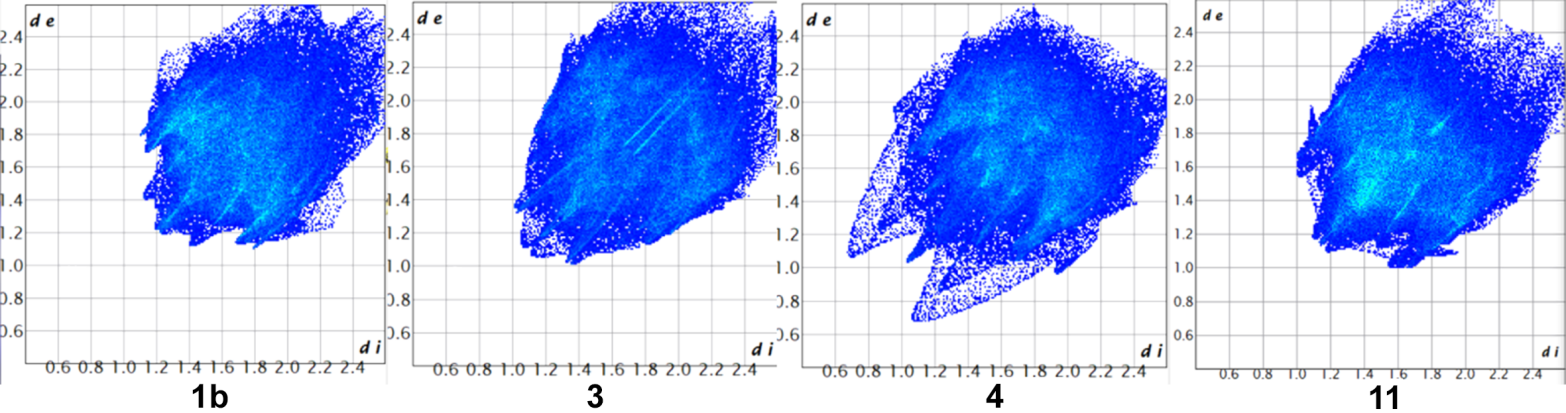
Fingerprint plots of **1b**, **3**, **4** and **11** (from left to right).

In order to get a better overview of the inter­molecular inter­actions at work, we undertook a Hirshfeld analysis, using the program *CrystalExplorer* (Spackman *et al.*, 2021 ▸). First, we determined the Hirshfeld surfaces (Fig. 9 ▸) and fingerprint plots (Fig. 10 ▸).

Evaluation of the fingerprint plots allowed the calculation of the relative contributions of inter­actions of elements inside and outside the Hirshfeld surface (Table 3 ▸).

As can be seen, inter­actions between hydrogen and other elements make up for more than 95% of all inter­actions and *ca* a 50% contribution comes from H⋯H inter­actions. This dominance is in part due to the large number of C—H bonds present (22 in **1b**, 23 in **3**, 25 in **4** and 30 in **11**) in relation to the number of Br and S atoms. For com­parison, in [Fe{C_5_(SMe)_5_}(C_5_H_5_)] (Blockhaus *et al.*, 2019 ▸), the inter­actions with H-atom contributions make up 97%, with 63.4% coming from H⋯H inter­actions alone (20 C—H bonds). However, S⋯S inter­actions contribute *ca* 3%. In [Mn{C_5_(SMe)_5_}(CO)_3_] (Sünkel & Motz, 1988 ▸), inter­actions with H-atom contributions make up *ca* 88%, with 34.8% coming from H⋯H and 24.6% from H⋯S; S⋯S inter­actions contribute 2.3%.

**Figure 11:**
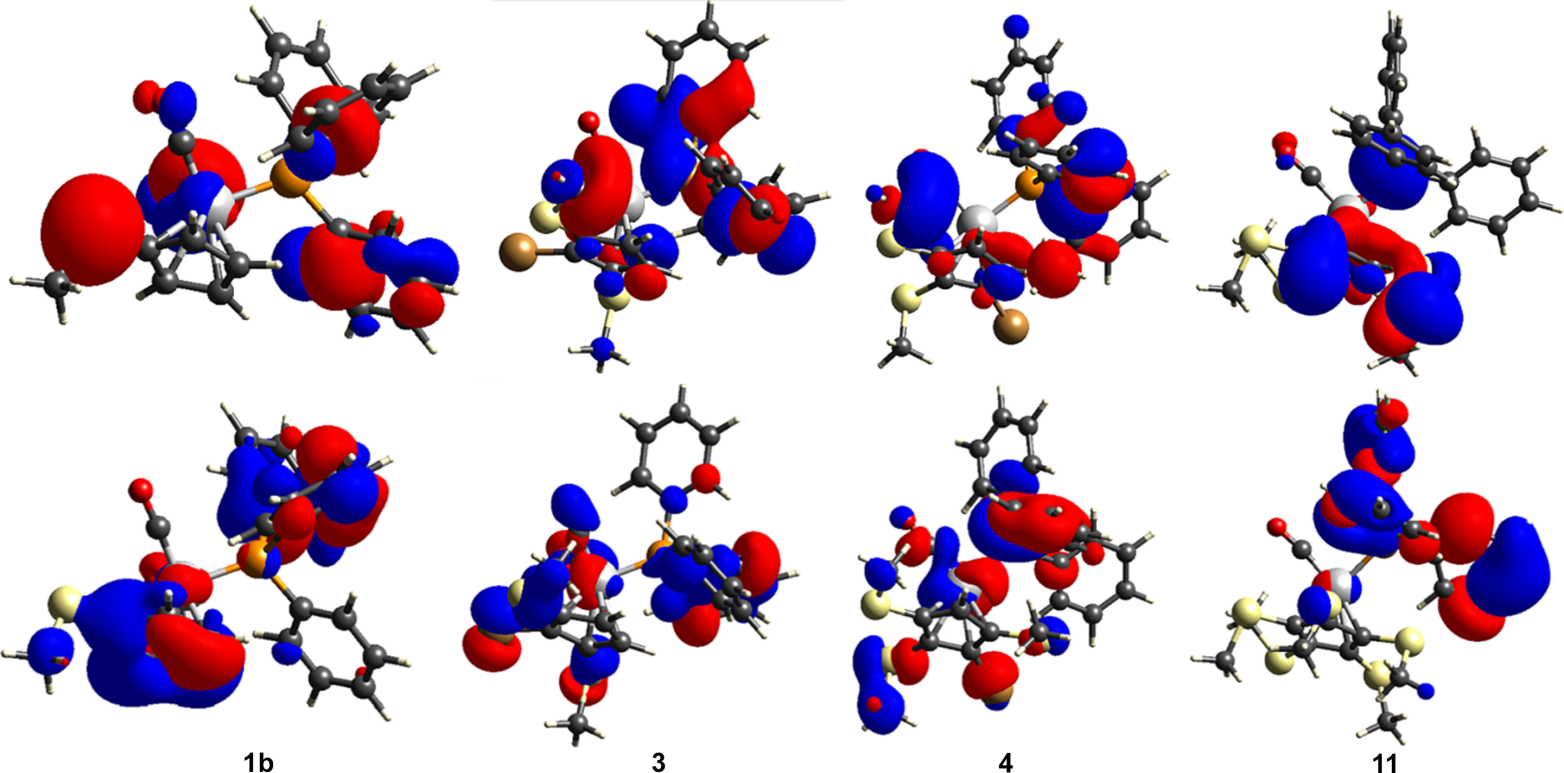
HOMO (top row) and LUMO (bottom row) of com­pounds **1b**, **3, 4** and **11** (from left to right).

We also performed an orbital calculation of the highest occupied mol­ecular orbital (HOMO) and lowest unoccupied mol­ecular orbital (LUMO) of com­pounds **1b**, **3**, **4** (major com­ponent) and **11**, using the program *TONTO* (HF/STO-3G), as provided with *CrystalExplorer*. The results are shown in Fig. 11 ▸.

As can be seen, for **1b**, the HOMO resides on the S atom, the CO ligands and one arene ring, while the LUMO is concentrated on the cyclo­penta­dienyl ring. In **3**, the HOMO is distributed over the CO ligands and the P atom and part of the arene rings, while the LUMO is mainly situated on the Br atom and the SMe groups. In com­pound **4** (major com­ponent), the HOMO resides mainly on two SMe groups, as well as on one arene ring. The LUMO is spread over the metal, the remaining SMe group and the Br atom, as well as on one other arene ring. For com­pound **11**, the HOMO resides on the Cp ring, including two SMe groups and the P atom, while the LUMO is distributed over the Mn atom and the PPh_3_ ligand.

## Conclusions

The best approach for the synthesis of [Mn{C_5_(SMe)_5_}(PPh_3_)(CO)_2_)] appears to be the one-pot reaction of [Mn(C_5_Br_5_)(PPh_3_)(CO)_2_] first with 2 equivalents of *n*-BuLi/MeSSMe and then with four equivalents of these reagents. Stepwise reactions, as well as the bottom-up approach starting with [Mn(C_5_H_4_Br)(PPh_3_)(CO)_2_], lead only to com­plicated product mixtures, which need multiple purification steps with large losses in yield. The structures of **1b**, **3**, **4** and **11** with one, two, three or five SMe groups, respectively, show an unpredictable distribution of axial and equatorial methyl groups. S⋯Br contacts in **3** leads to the formation of ‘dimers’, while S⋯S contacts in **11** lead to polymeric one-dimensional strands. Besides these, no structure-directing effects of halogen or chalcogen (S) bonding can be observed.

## Supplementary Material

Crystal structure: contains datablock(s) compd_1b, compd_3, compd_4, compd_11, comp_2, global. DOI: 10.1107/S205322962400603X/qf3063sup1.cif

Structure factors: contains datablock(s) compd_1b. DOI: 10.1107/S205322962400603X/qf3063compd_1bsup2.hkl

Structure factors: contains datablock(s) compd_3. DOI: 10.1107/S205322962400603X/qf3063compd_3sup3.hkl

Structure factors: contains datablock(s) compd_4. DOI: 10.1107/S205322962400603X/qf3063compd_4sup4.hkl

Structure factors: contains datablock(s) compd_11. DOI: 10.1107/S205322962400603X/qf3063compd_11sup5.hkl

Structure factors: contains datablock(s) comp_2. DOI: 10.1107/S205322962400603X/qf3063comp_2sup6.hkl

Supporting information file. DOI: 10.1107/S205322962400603X/qf3063sup7.pdf

CCDC references: 2364358, 2364357, 2364356, 2364355, 2364354

## Figures and Tables

**Table:** Experiments were carried out with Mo *K*α radiation using a Bruker D8 VENTURE diffractometer. Absorption was corrected for by multi-scan methods (*SADABS2016*; Krause *et al.*, 2015 ▸). H-atom parameters were constrained.

	**1b**	**3**	**4**
Crystal data			
Chemical formula	[Mn(C_6_H_7_S)(C_18_H_15_P)(CO)_2_]	[Mn(C_7_H_8_BrS_2_)(C_18_H_15_P)(CO)_2_]	[Mn(C_8_H_10_BrS)(C_18_H_15_P)(CO)_2_]
*M*_r_	484.40	609.39	655.48
Crystal system, space group	Monoclinic, *P*2_1_/*c*	Orthorhombic, *Pbca*	Monoclinic, *P*2_1_/*n*
Temperature (K)	297	108	108
*a*, *b*, *c* (Å)	7.7281 (3), 16.8523 (6), 18.0339 (7)	16.5563 (7), 16.2392 (7), 19.3372 (9)	8.7717 (4), 13.2135 (6), 23.9428 (12)
α, β, γ (°)	90, 96.696 (1), 90	90, 90, 90	90, 92.884 (2), 90
*V* (Å^3^)	2332.65 (15)	5199.0 (4)	2771.6 (2)
*Z*	4	8	4
μ (mm^−1^)	0.74	2.29	2.23
Crystal size (mm)	0.10 × 0.03 × 0.03	0.06 × 0.03 × 0.03	0.07 × 0.03 × 0.02

Data collection			
*T*_min_, *T*_max_	0.702, 0.745	0.691, 0.745	0.676, 0.746
No. of measured, independent and observed [*I* > 2σ(*I*)] reflections	46361, 4773, 4190	71764, 5317, 4408	51088, 6886, 5787
*R*_int_	0.036	0.070	0.047
(sin θ/λ)_max_ (Å^−1^)	0.626	0.625	0.667

Refinement			
*R*[*F*^2^ > 2σ(*F*^2^)], *wR*(*F*^2^), *S*	0.028, 0.081, 1.05	0.035, 0.088, 1.07	0.033, 0.071, 1.10
No. of reflections	4773	5317	6886
No. of parameters	281	309	359
No. of restraints	0	0	13
Δρ_max_, Δρ_min_ (e Å^−3^)	0.28, −0.26	1.75, −0.83	0.44, −0.44

**Table:** 

	**11**	**2**
Crystal data		
Chemical formula	[Mn(C_10_H_15_S)(C_18_H_15_P)(CO)_2_]	[Mn(C_6_H_6_SBr)(C_18_H_15_P)(CO)_2_]·0.75C_6_H_12_
*M*_r_	668.75	1189.73
Crystal system, space group	Monoclinic, *P*2_1_/*c*	Triclinic, *P* 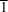
Temperature (K)	107	108
*a*, *b*, *c* (Å)	12.5274 (5), 13.6748 (5), 17.9841 (7)	10.3309 (5), 10.7727 (5), 13.0821 (6)
α, β, γ (°)	90, 107.934 (1), 90	87.692 (2), 82.138 (2), 62.507 (2)
*V* (Å^3^)	2931.2 (2)	1278.93 (11)
*Z*	4	1
μ (mm^−1^)	0.89	2.25
Crystal size (mm)	0.03 × 0.03 × 0.02	0.05 × 0.03 × 0.02

Data collection		
*T*_min_, *T*_max_	0.714, 0.746	0.691, 0.746
No. of measured, independent and observed [*I* > 2σ(*I*)] reflections	46511, 6471, 5457	21220, 6351, 5421
*R*_int_	0.056	0.030
(sin θ/λ)_max_ (Å^−1^)	0.641	0.667

Refinement		
*R*[*F*^2^ > 2σ(*F*^2^)], *wR*(*F*^2^), *S*	0.030, 0.076, 1.04	0.066, 0.170, 1.03
No. of reflections	6471	6351
No. of parameters	357	328
No. of restraints	0	2
Δρ_max_, Δρ_min_ (e Å^−3^)	0.41, −0.50	5.86, −1.71

Computer programs: *APEX2* (Bruker, 2011 ▸), *SAINT* (Bruker, 2011 ▸), *SHELXT2014* (Sheldrick, 2015*a* ▸), *SHELXL2018* (Sheldrick, 2015*b* ▸) and *Mercury* (Macrae *et al.*, 2020 ▸).

**Table 2:** Important bond lengths (Å) and angles (°) in **1b**, **3**, **4** and **11**

	**1b**	**3**	**4**	**11**
Mn—P	2.2408 (5)	2.2413 (8)	2.2565 (7)	2.2660 (6)
Mn—CO	1.770 (2)	1.770 (3)	1.788 (3)	1.786 (1)
	1.767 (2)	1.766 (3)	1.774 (2)	1.776 (2)
Mn—Ct_Cp_	1.772 (1)	1.770 (1)	1.792 (1)	1.785 (1)
C_Cp_—Br	–	1.874 (3)	1.871 (3)*	–
C_cp_—S, average	1.766 (2)	1.755 (2)	1.748 (3)	1.763 (2)
S—C_Me_, average	1.794 (3)	1.786 (4)	1.802 (3)	1.811 (2)
Mn⋯S	3.4778 (6)	3.564 (1)	3.590 (1)/3.551 (1)	3.5157 (7)–
		3.465 (1)	3.47 (1)	3.5880 (6)
C_Cp_—S—C_Me_	99.0 (1)	102.4 (2)	100.5 (1)	98.9 (1)–
		99.4 (2)	101.6 (1)	104.0 (1)
C—C_Cp_—S—C_Me_	92.0 (2)	19.9 (3)	12.8 (3)/26.4 (3)	50.6 (2)–
		88.0 (3)	83.1 (3)	68.4 (2)
S—Ct_Cp_—Mn—P	158.9	91.8	127.6/19.3	Meaningless
		127.0	−161.8	

Note: (*) major com­ponent.

**Table 3:** Relative contributions (%) of elements to the inter­actions of atoms inside and outside the Hirshfeld surface (mainly contributions > 2%)

	**1b**	**3**	**4**	**11**
H⋯H	49.5	42.5	45.2	55.4
H⋯C	22.9	22.4	20.8	15.4
H⋯O	19.4	15.8	15.1	12.0
H⋯S	6.4	8.0	10.5	14.8
H⋯Br	–	8.4	7.2	–
Br⋯S	–	2.1	0.2	–
S⋯S	0.0	0.0	0.3	0.6
C⋯C	1.4	0.3	0.0	0.4
