# Supplementary material for: Synthesis, spectroscopic and crystallographic characterization of various cymantrenyl thio­ethers [Mn{C5HxBry(SMe)z}(PPh3)(CO)2]
Source: Acta Crystallogr C Struct Chem. 2024 Jul 5;80(Pt 8):383–93. doi: 10.1107/S205322962400603X (PMC11299206; doi:10.1107/S205322962400603X)
Supplement: Supplementary file 7 [file c-80-00383-sup7.pdf]

Supplementary Information

Inhalt

1. NMR Spectra 4

2. Mass spectra 17

3. Crystal and Molecular Structures 19

Figure S 1 ^{1}H NMR spectrum of **1b** 4
Figure S 2 $^{13}C\{^{1}H\}$ NMR spectrum of **1b** 4
Figure S 3 ^{31}P NMR spectrum of **1b** 5
Figure S 4 ^{1}H NMR spectrum of **2** 5
Figure S 5 $^{13}C\{^{1}H\}$ NMR spectrum of **2** 6
Figure S 6 ^{31}P NMR spectrum of **2** 6
Figure S 7 ^{1}H NMR spectrum of **3** 7
Figure S 8 $^{13}C\{^{1}H\}$ NMR spectrum of **3** 7
Figure S 9 ^{31}P NMR spectrum of **3** 8
Figure S 10 ^{1}H NMR spectrum of crude product from the reaction of **3** with 2.5 eq. LiTMP and Me_2S_2 8
Figure S 11 ^{31}P NMR spectrum of crude product from the reaction of **3** with 2.5 eq. LiTMP and Me_2S_2 9
Figure S 12 ^{31}P NMR spectrum of fraction F1 of the chromatographic separation of the reaction product of **3** with 2.5 eq. LiTMP and Me_2S_2 9
Figure S 13 ^{1}H NMR spectrum of fraction F2.1 of the chromatographic separation of the reaction product of **3** with 2.5 eq. LiTMP and Me_2S_2 : Nearly pure **4**. 10
Figure S 14 ^{31}P NMR spectrum of fraction F2.1 of the chromatographic separation of the reaction product of **3** with 2.5 eq. LiTMP and Me_2S_2: Nearly pure **4** 10
Figure S 15 ^{1}H NMR spectrum of fraction F2.2 of the chromatographic separation of the reaction product of **3** with 2.5 eq. LiTMP and Me_2S_2 11
Figure S 16: Partial $^{31}P\{^{1}H\}$ NMR spectrum of fraction F2.2 of the chromatographic separation of the reaction product of **3** with 2.5 eq. LiTMP and Me_2S_2 11
Figure S 17 ^{1}H NMR spectrum (400 MHz, $CDCl_3$) of the crude reaction mixture obtained from the reaction of **6** with 2.5 eq. n-BuLi/S_2Me_2 in THF at -78°C 12
Figure S 18 $^{31}P\{^{1}H\}$ NMR spectrum (162 MHz, $CDCl_3$) of the crude reaction mixture obtained from the reaction of **6** with 2.5 eq. n-BuLi/S_2Me_2 in THF at -78°C 12
Figure S 19 ^{1}H NMR spectrum (400 MHz, $CDCl_3$) of (impure) **7** 13
Figure S 20 $^{31}P\{^{1}H\}$ NMR spectrum (400 MHz, $CDCl_3$) of (impure) **7** 13
Figure S 21 $^{13}C\{^{1}H\}$ NMR spectrum (101 MHz, $CDCl_3$) of (impure) **7** 14
Figure S 22 ^{1}H NMR spectrum (400 MHz, $CDCl_3$) of the reaction product of a mixture of **7-10** with two consecutive additions of BuLi/ MeSSMe. 14
Figure S 23: $^{31}P\{^{1}H\}$ NMR spectrum (161 MHz, $CDCl_3$) of the reaction product of a mixture of **7-10** with two consecutive additions of BuLi/ MeSSMe. 15
Figure S 24 ^{1}H-NMR spectrum (400 MHz, $CDCl_3$) of **11** 15
Figure S 25 $^{31}P\{^{1}H\}$-NMR spectrum (162 MHz, $CDCl_3$) of **11** 16
Figure S 26 $^{13}C\{^{1}H\}$-NMR spectrum (101 MHz, $CDCl_3$) of **11** 16
Figure S 27 DEI-MS of compound **1b** 17
Figure S 28 DEI-MS of crude product from the reaction of **3** with 2.5 eq. LiTMP and Me_2S_2 17
Figure S 29 DEI MS of the crude reaction mixture obtained from the reaction of **6** with 2.5 eq. n-BuLi/S_2Me_2 in THF at -78°C 18
Figure S 30 DEI mass spectrum of **11** 18

Figure S 31: Top and side view of the molecular structure of compound **2**. 19

1. NMR Spectra

General remark: Identified impurities in the spectra are marked as follows: C (chloroform), M (methylene chloride), E (diethyl ether), W (water), PE (petroleum ether), S (silicon grease), THF (tetrahydrofuran).

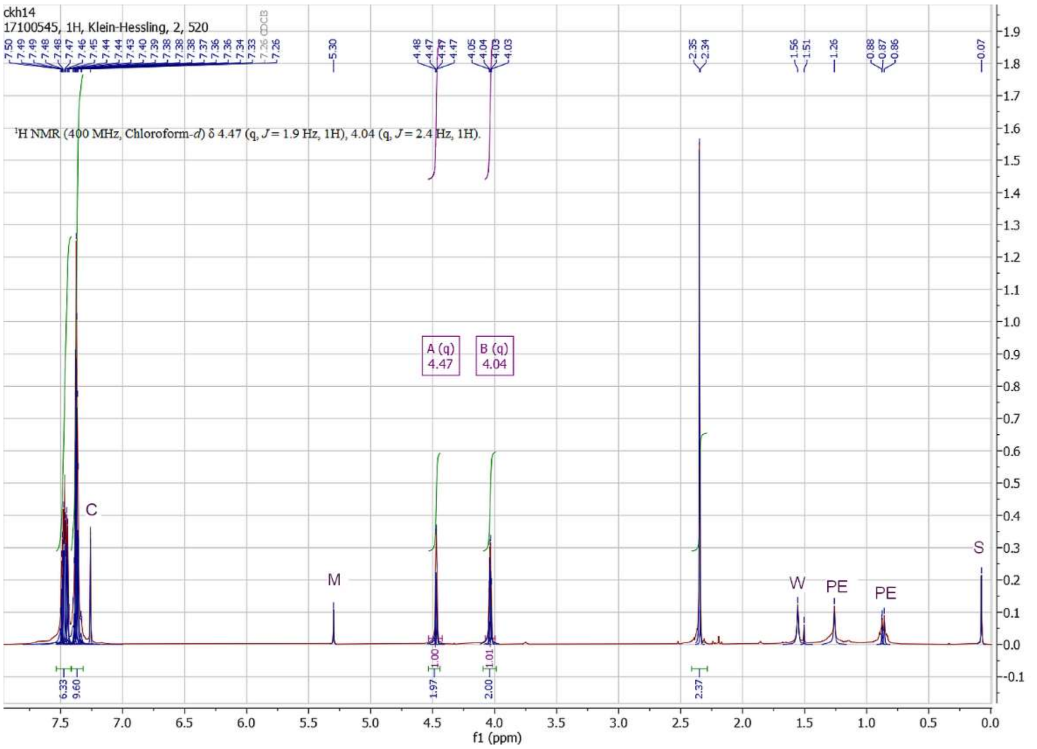

Figure S 1 ¹H NMR spectrum of **1b**

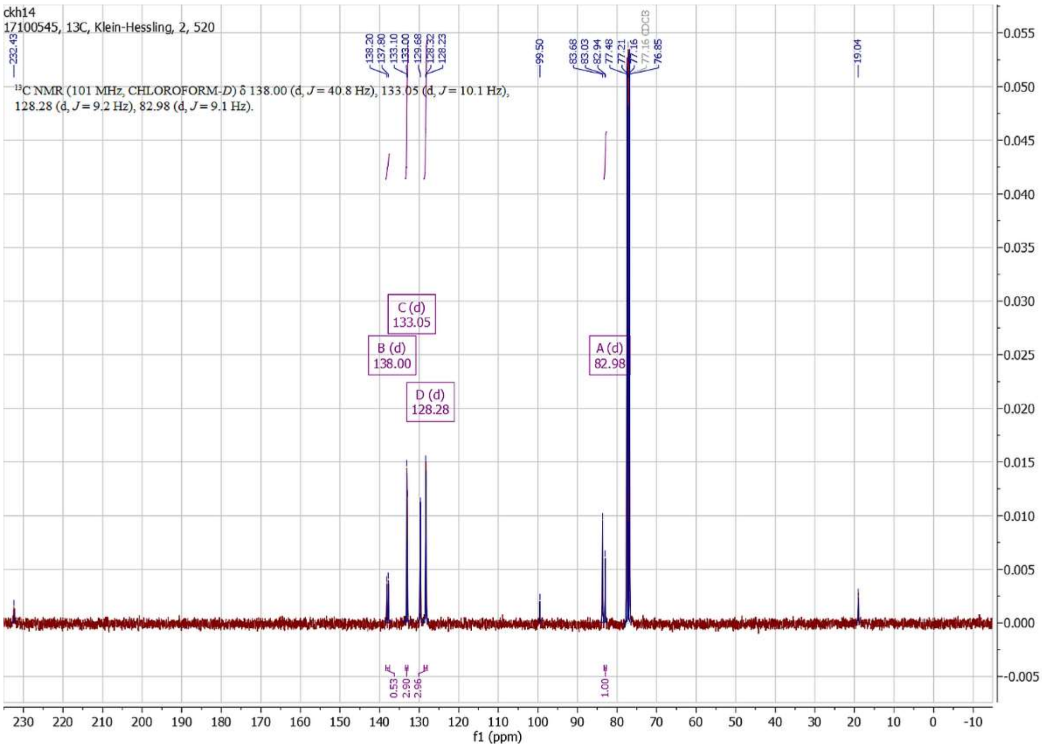

Figure S 2 ¹³C{¹H} NMR spectrum of **1b**

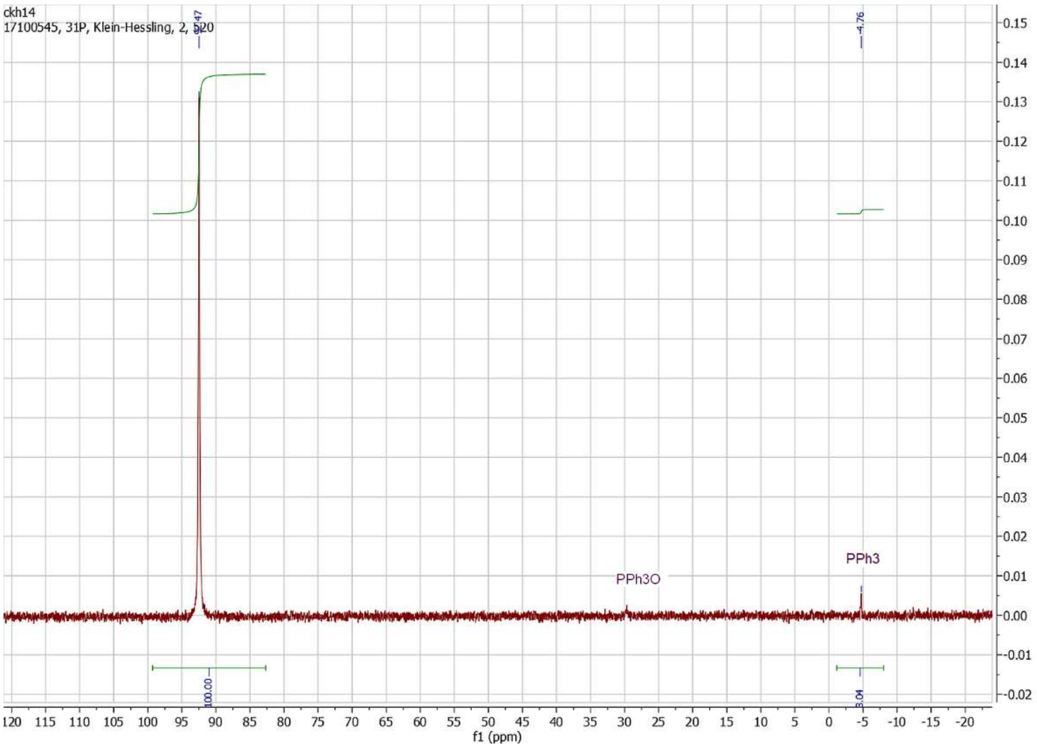

Figure S 3 ^{31}P NMR spectrum of **1b**

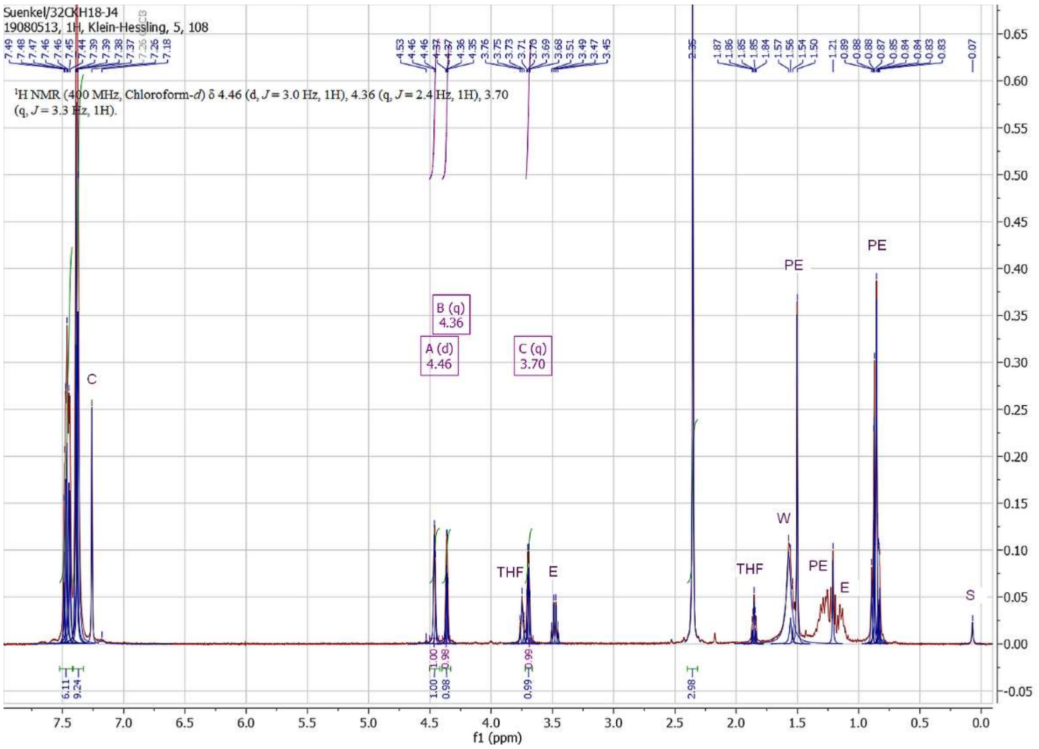

Figure S 4 ^{1}H NMR spectrum of **2**

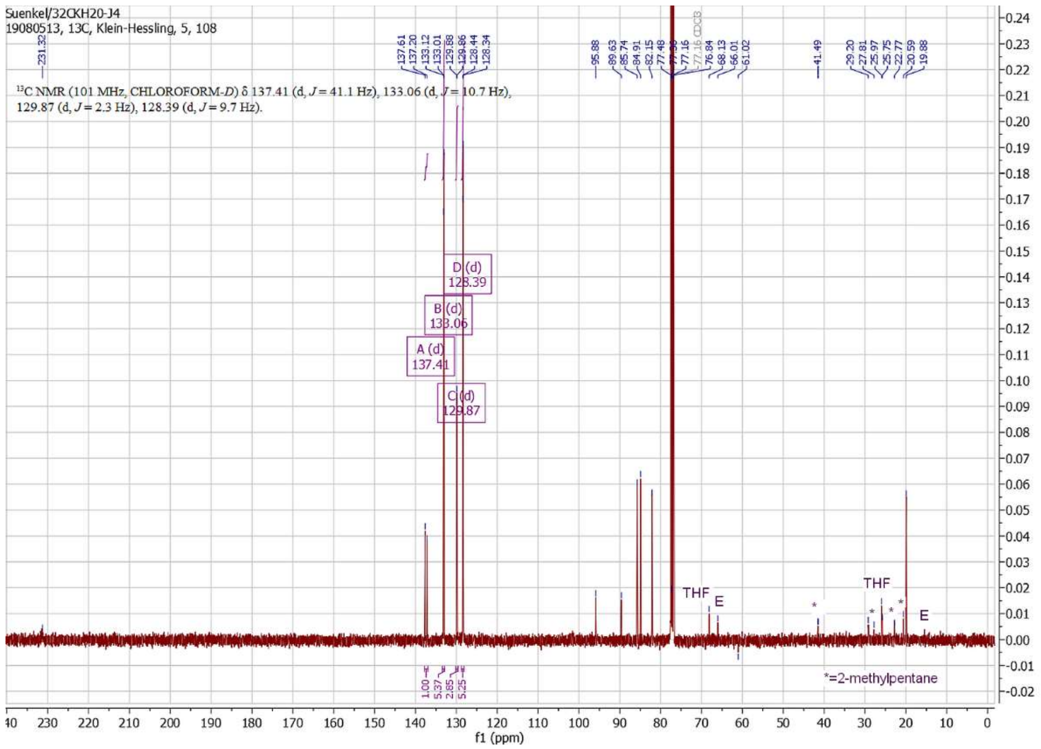

Figure S 5 $^{13}C\{^{1}H\}$ NMR spectrum of **2**

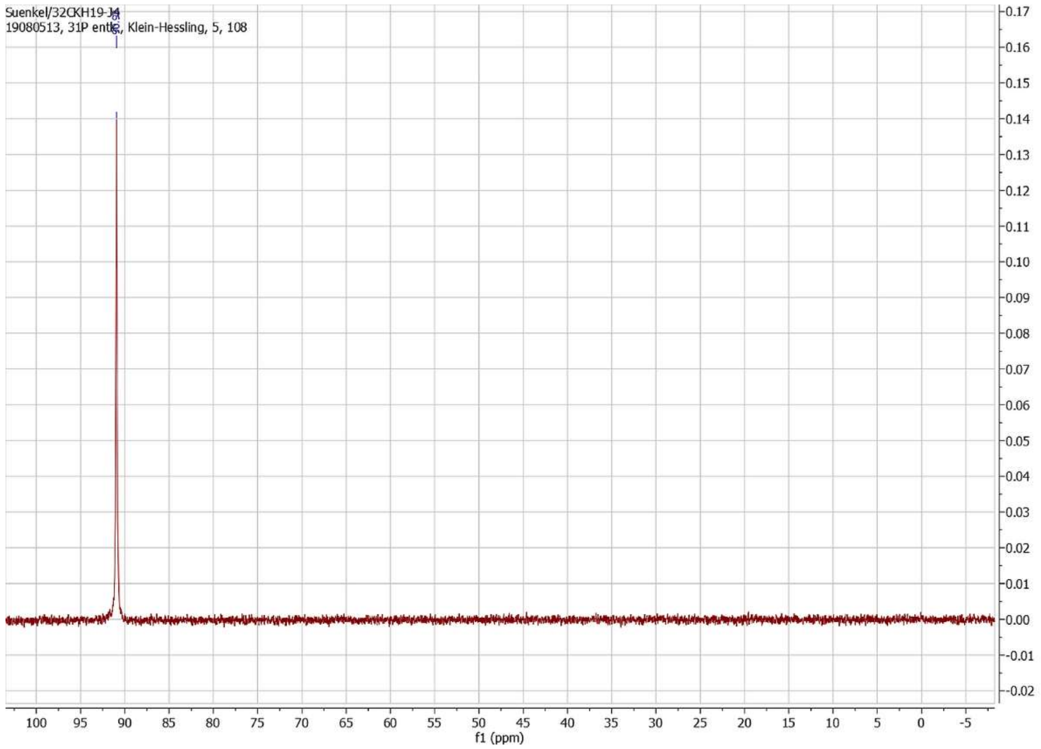

Figure S 6 ^{31}P NMR spectrum of **2**

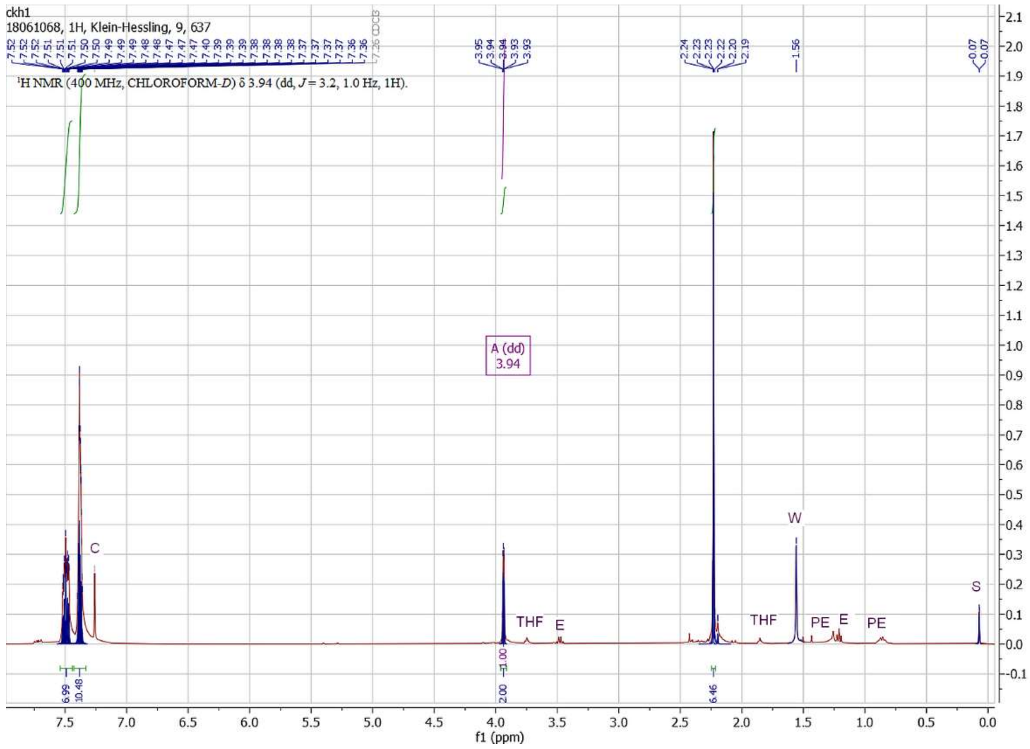

Figure S 7 ^{1}H NMR spectrum of **3**

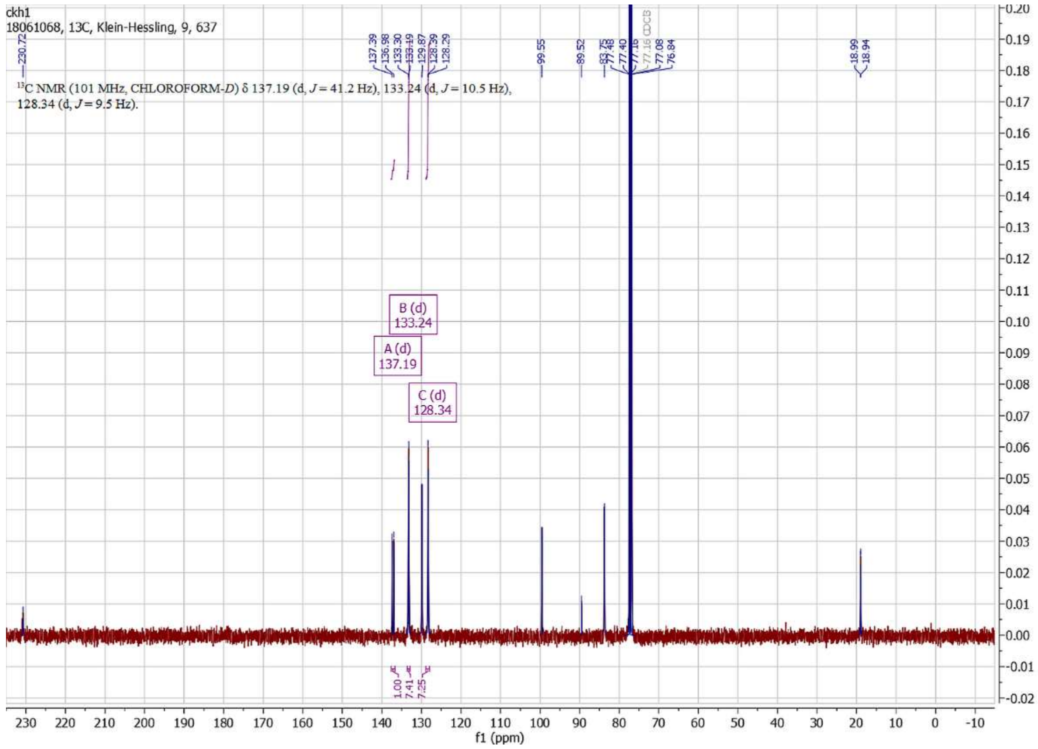

Figure S 8 ^{13}C{^{1}H} NMR spectrum of **3**

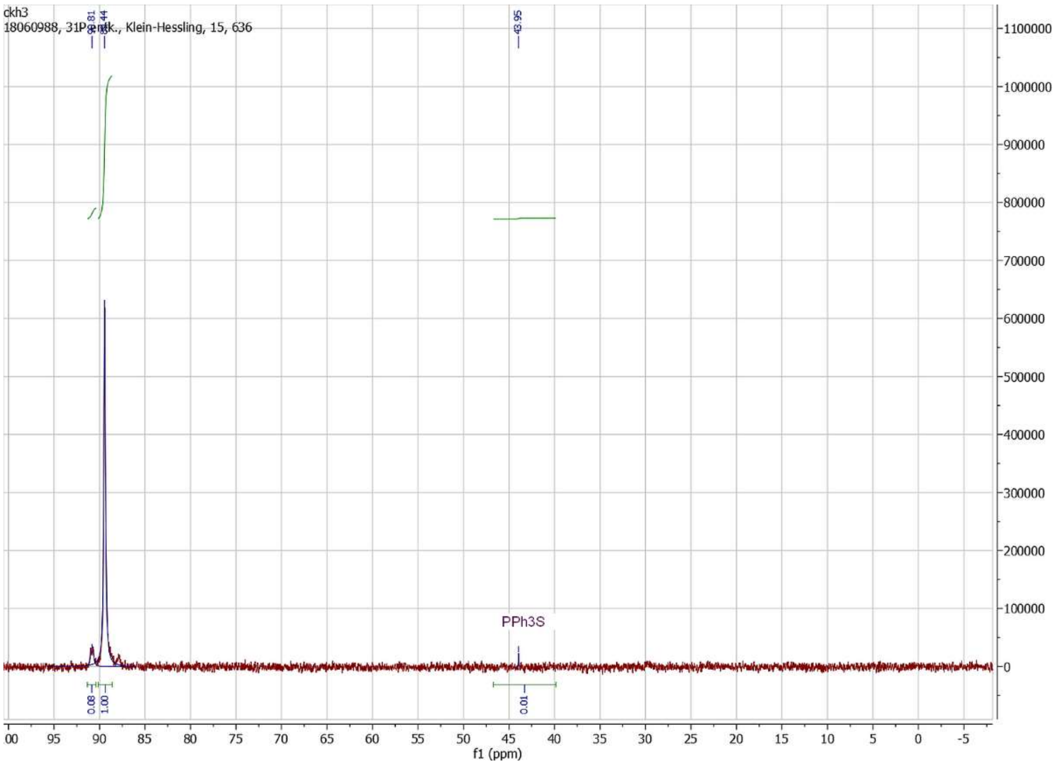

Figure S 9 ^{31}P NMR spectrum of **3**

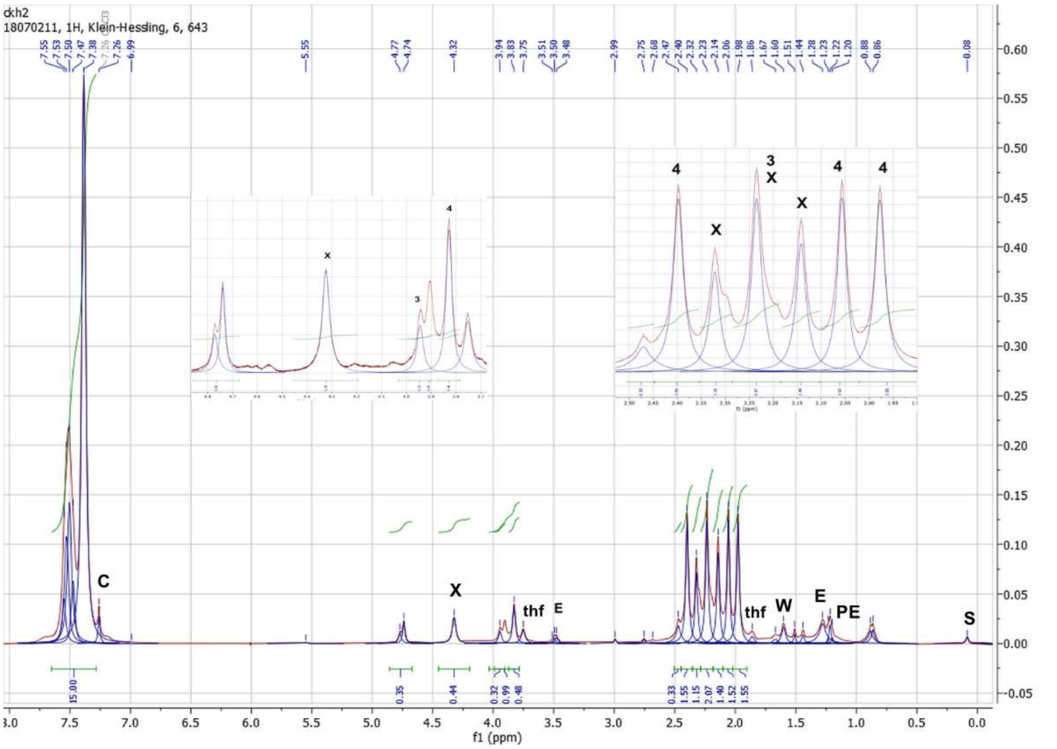

Figure S 10 ^{1}H NMR spectrum of crude product from the reaction of **3** with 2.5 eq. LiTMP and Me_2S_2

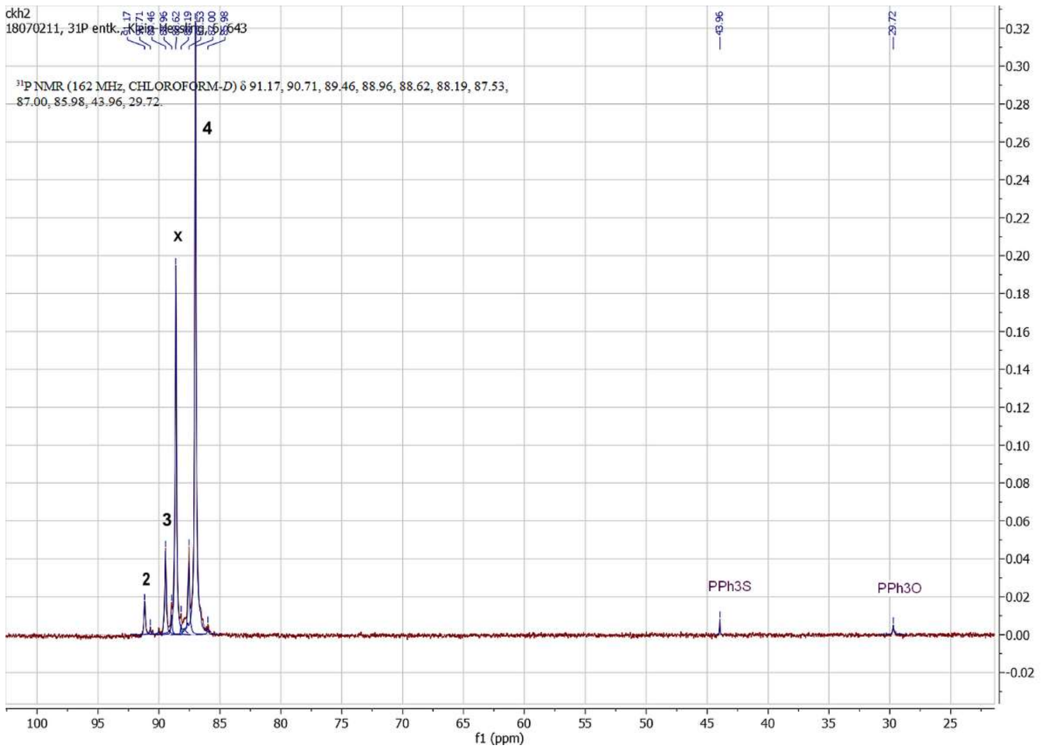

Figure S 11 ^{31}P NMR spectrum of crude product from the reaction of **3** with 2.5 eq. LiTMP and Me_2S_2

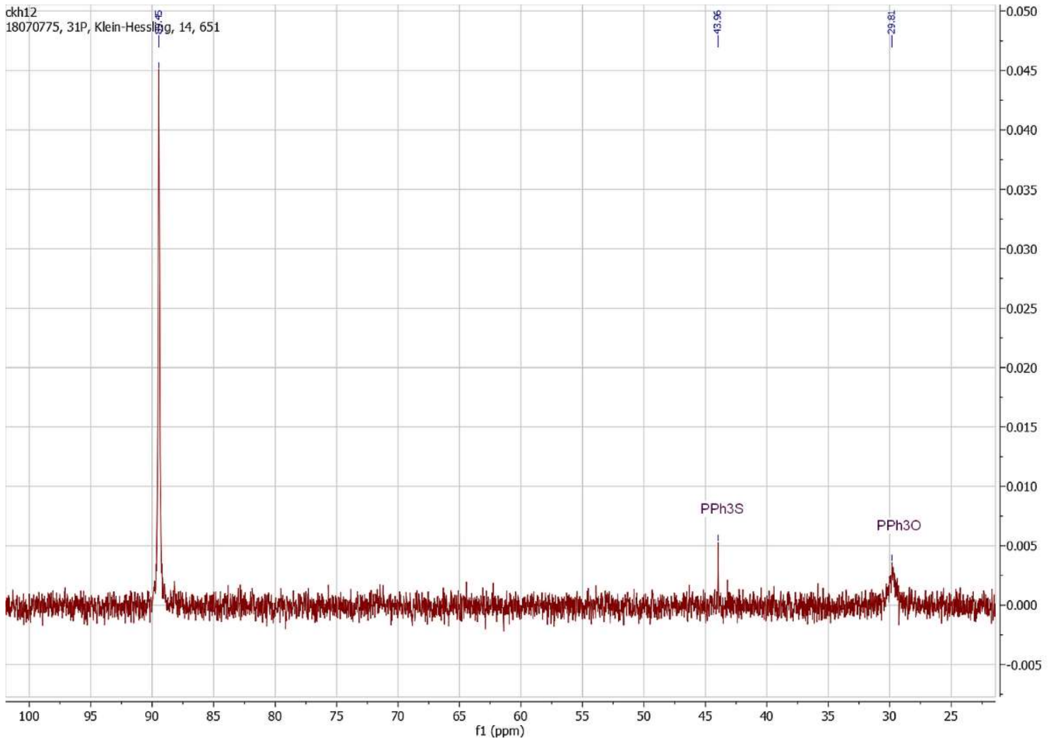

Figure S 12 ^{31}P NMR spectrum of fraction F1 of the chromatographic separation of the reaction product of **3** with 2.5 eq. LiTMP and Me_2S_2

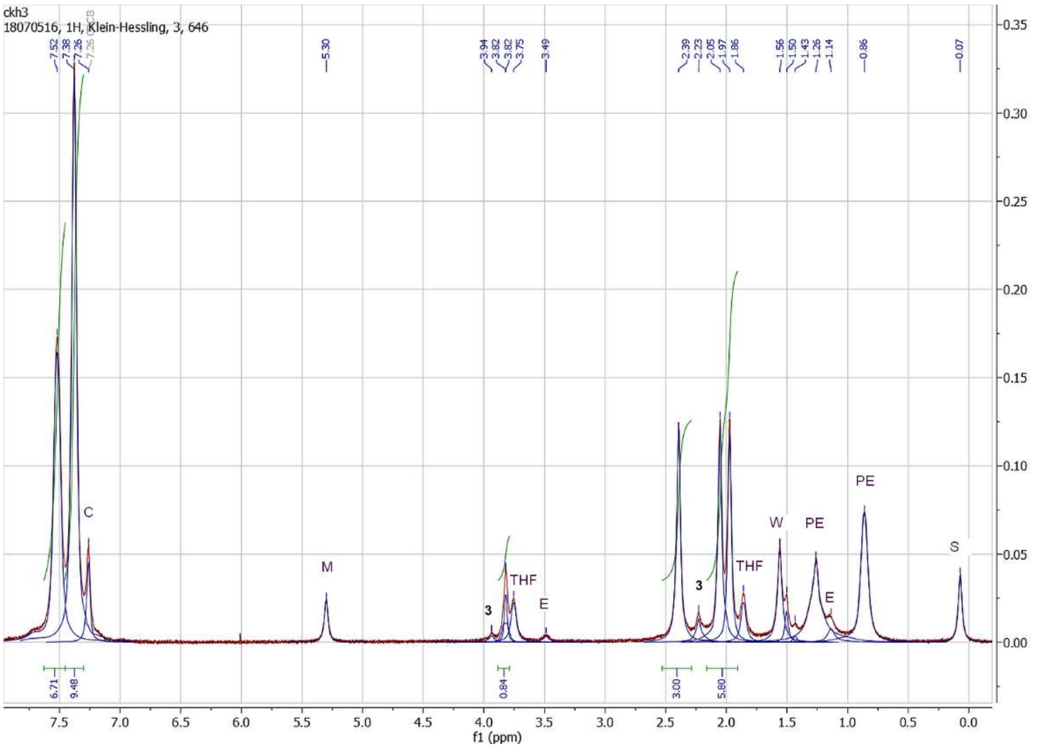

Figure S 13 ^{1}H NMR spectrum of fraction F2.1 *of the chromatographic separation of the reaction product* of **3** with 2.5 eq. LiTMP and Me_2S_2 : Nearly pure **4**.

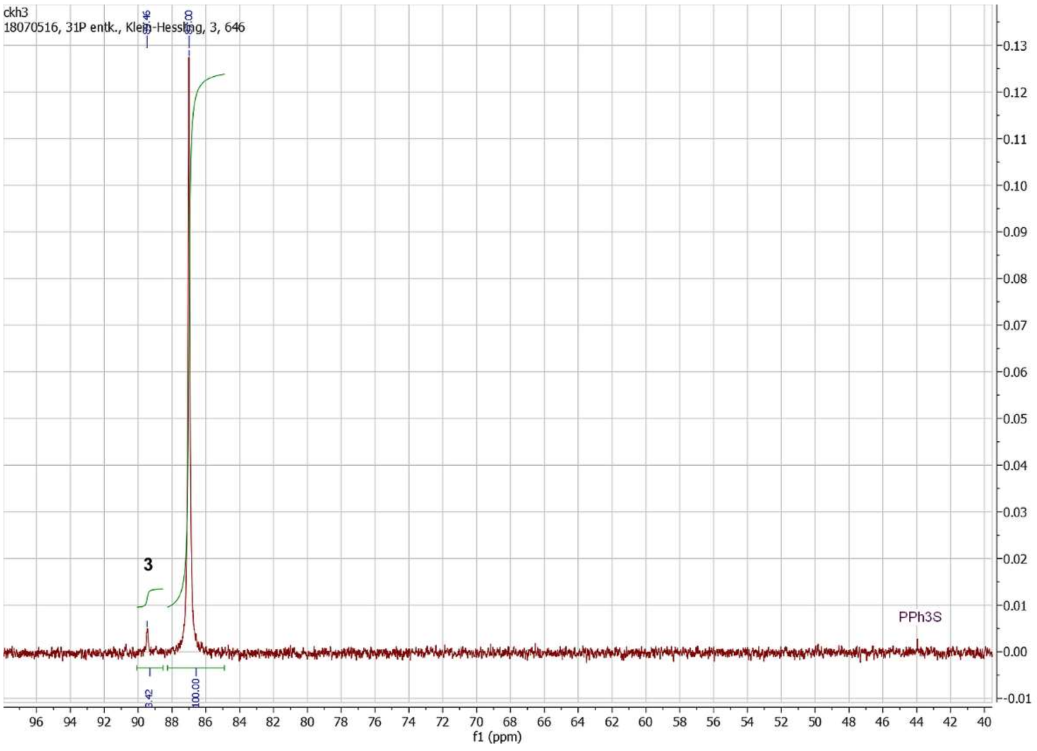

Figure S 14 ^{31}P NMR spectrum of fraction F2.1 *of the chromatographic separation of the reaction product* of **3** with 2.5 eq. LiTMP and Me_2S_2: Nearly pure **4**

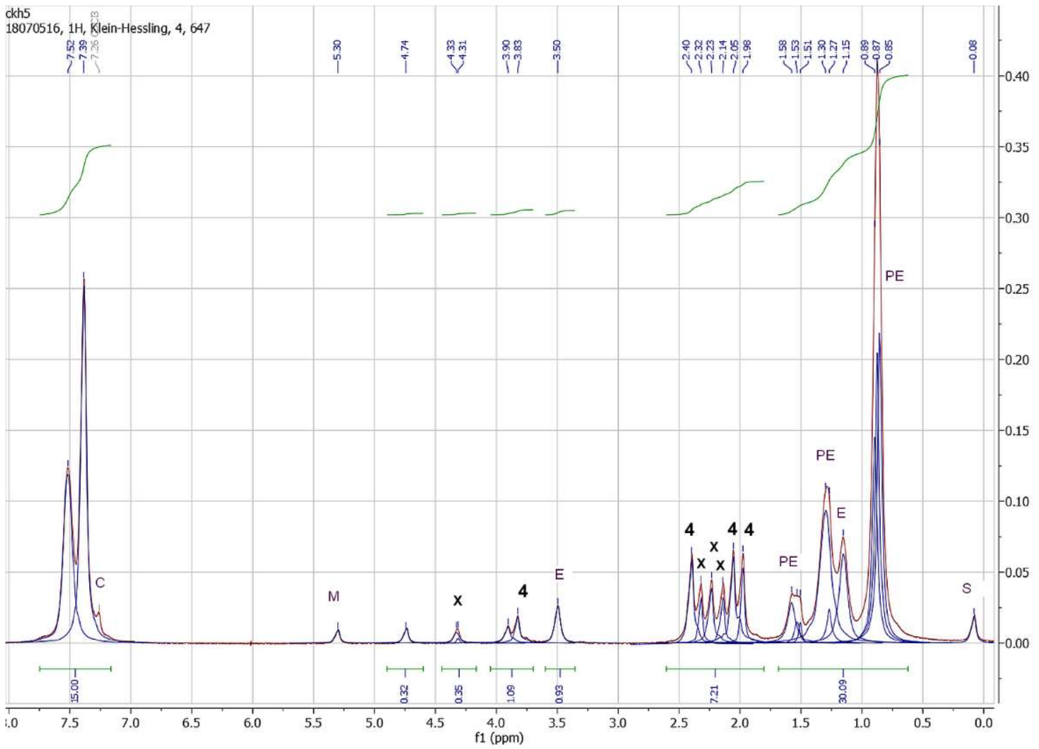

Figure S 15 ^{1}H NMR spectrum of fraction F2.2 of the chromatographic separation of the reaction product of **3** with 2.5 eq. LiTMP and Me_2S_2

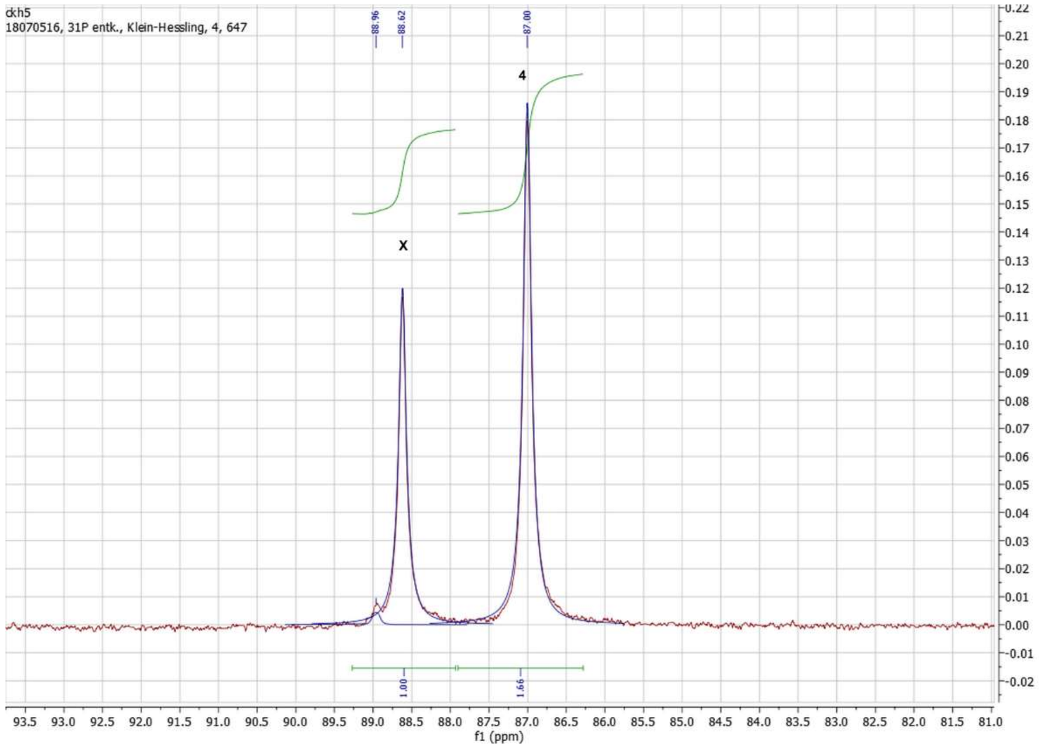

Figure S 16: Partial $^{31}P\{^{1}H\}$ NMR spectrum of fraction F2.2 of the chromatographic separation of the reaction product of **3** with 2.5 eq. LiTMP and Me_2S_2

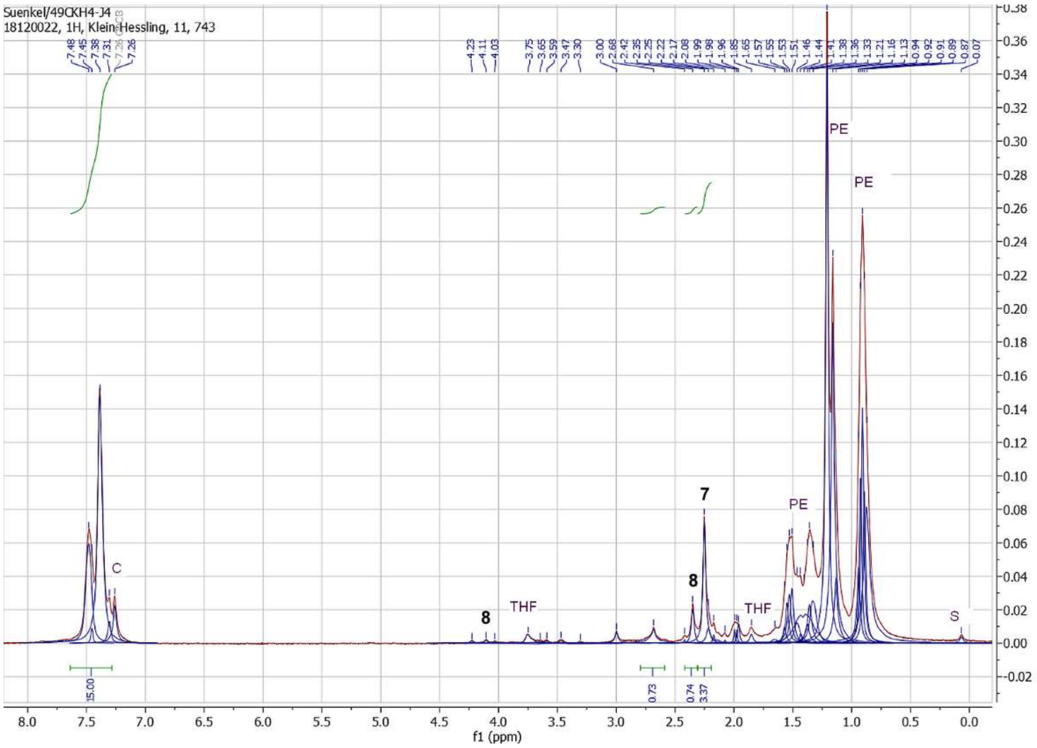

Figure S 17 ^{1}H NMR spectrum (400 MHz, $CDCl_3$) of the crude reaction mixture obtained from the reaction of **6** with 2.5 eq. n-BuLi/S_2Me_2 in THF at -78°C

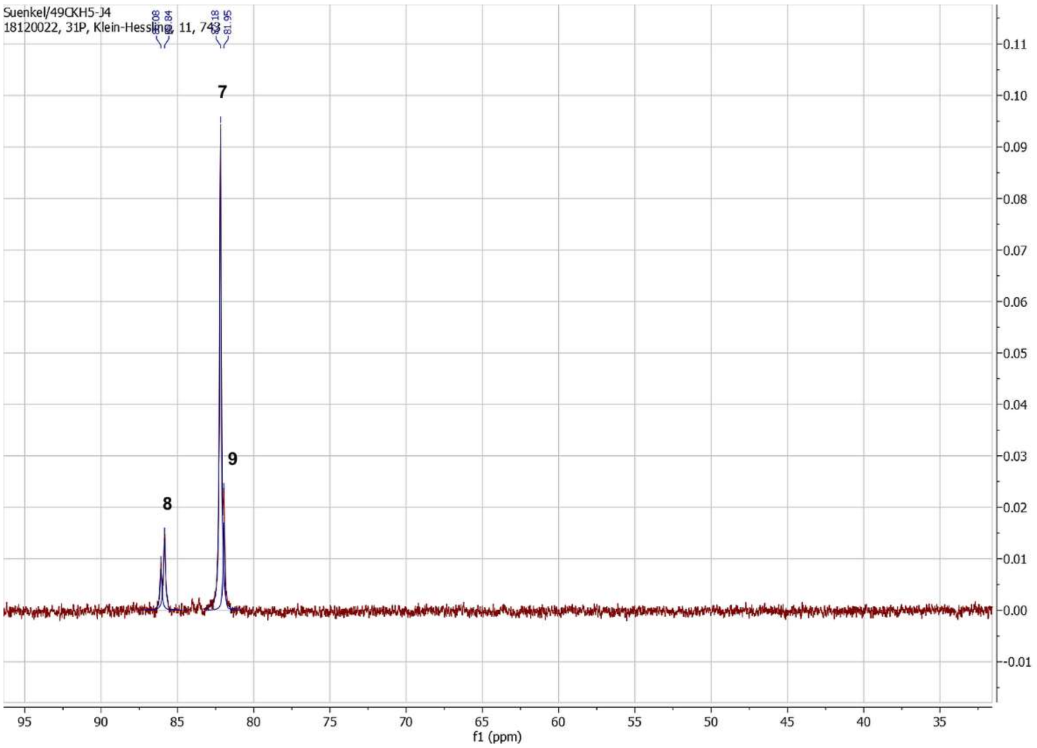

Figure S 18 $^{31}P\{^{1}H\}$ NMR spectrum (162 MHz, $CDCl_3$) of the crude reaction mixture obtained from the reaction of **6** with 2.5 eq. n-BuLi/S_2Me_2 in THF at -78°C

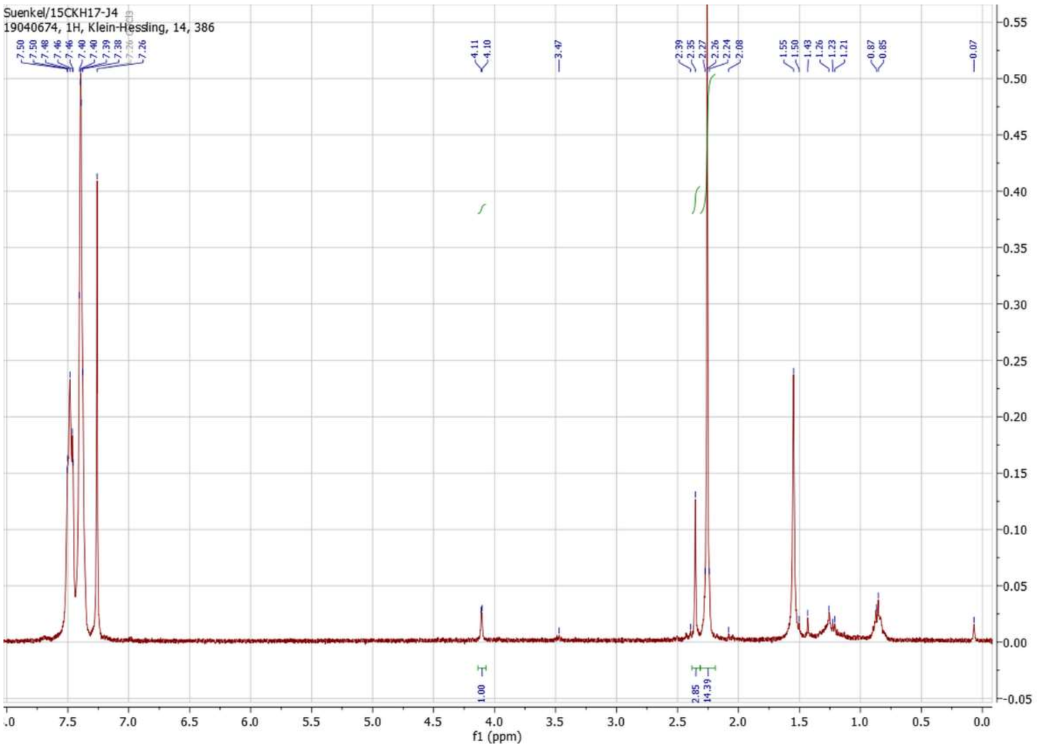

Figure S 19 ^{1}H NMR spectrum (400 MHz, $CDCl_3$) of (impure) **7**

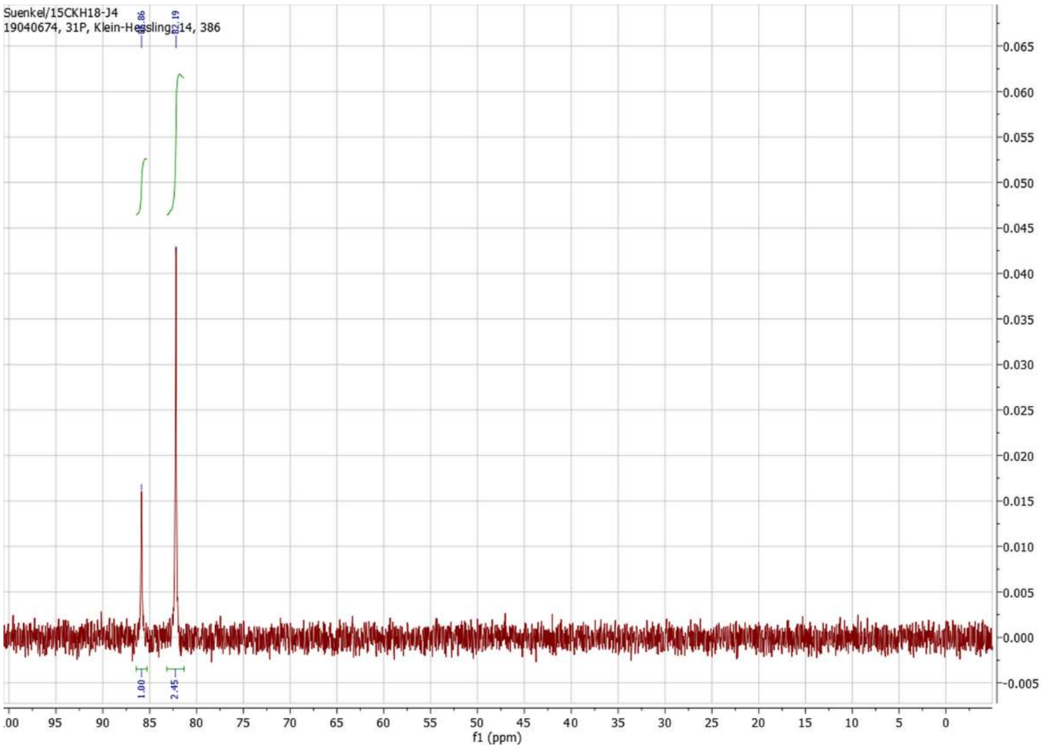

Figure S 20 $^{31}P\{^{1}H\}$ NMR spectrum (400 MHz, $CDCl_3$) of (impure) **7**

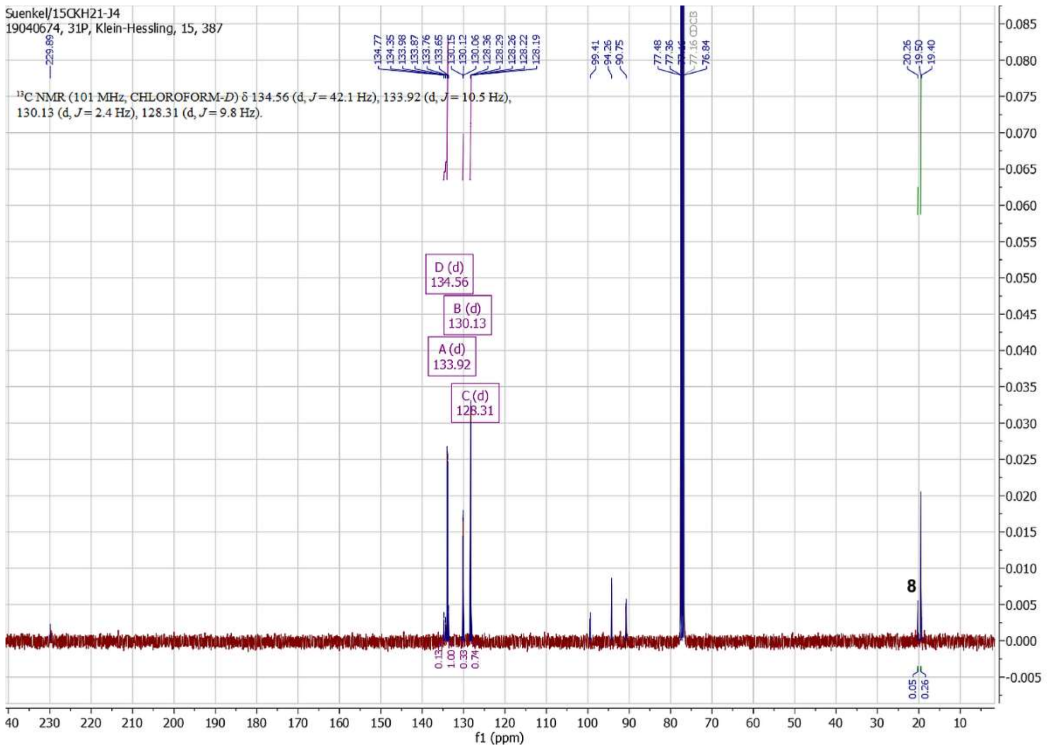

Figure S 21 $^{13}C\{^1H\}$ NMR spectrum (101 MHz, $CDCl_3$) of (impure) **7**

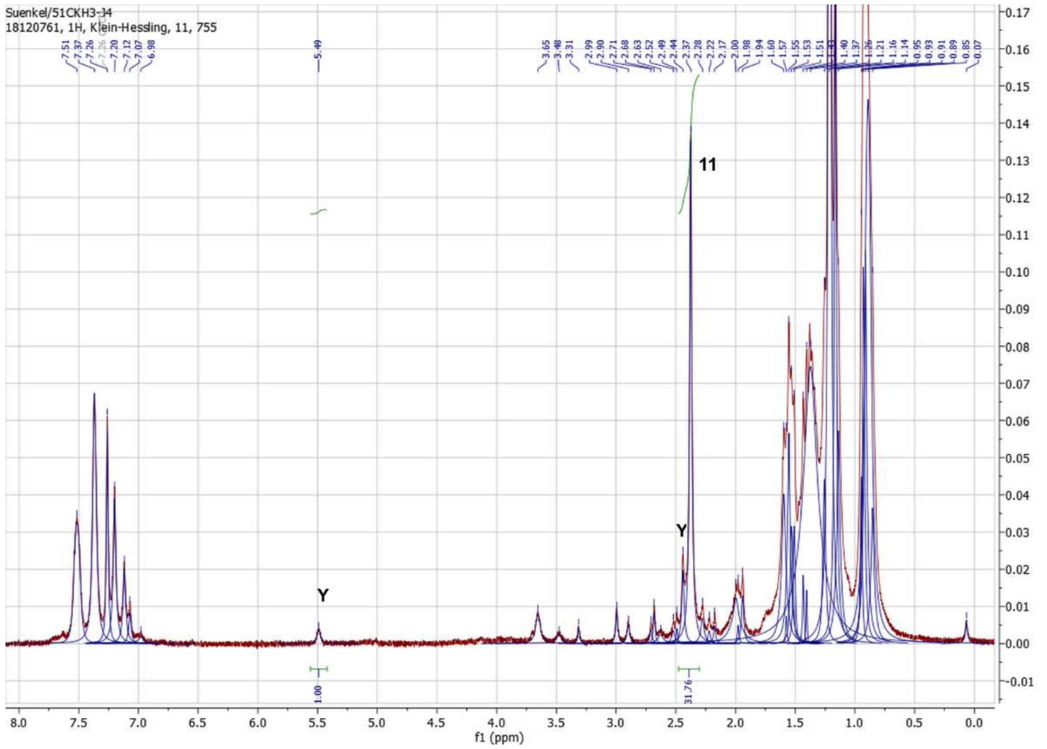

Figure S 22 ^1H NMR spectrum (400 MHz, $CDCl_3$) of the reaction product of a mixture of **7-10** with two consecutive additions of BuLi/ MeSSMe.

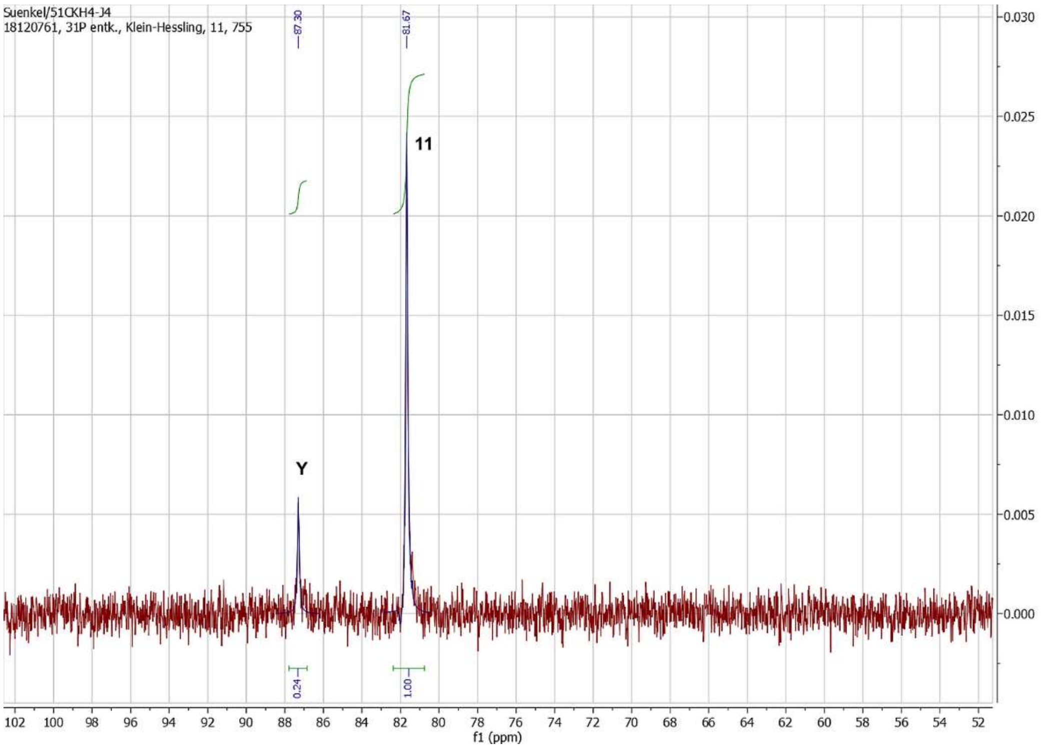

Figure S 23: ${}^{31}P\{{}^{1}H\}$ NMR spectrum (161 MHz, $CDCl_3$) of the reaction product of a mixture of **7-10** with two consecutive additions of BuLi/ MeSSMe.

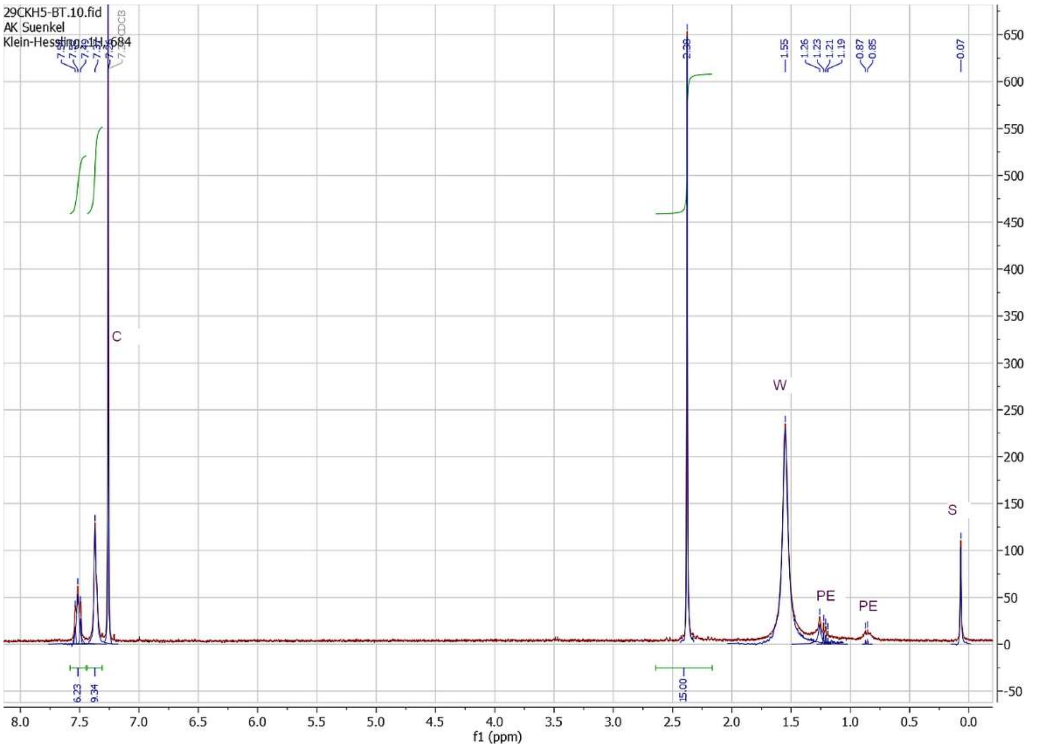

Figure S 24 ${}^{1}H$-NMR spectrum (400 MHz, $CDCl_3$) of **11**

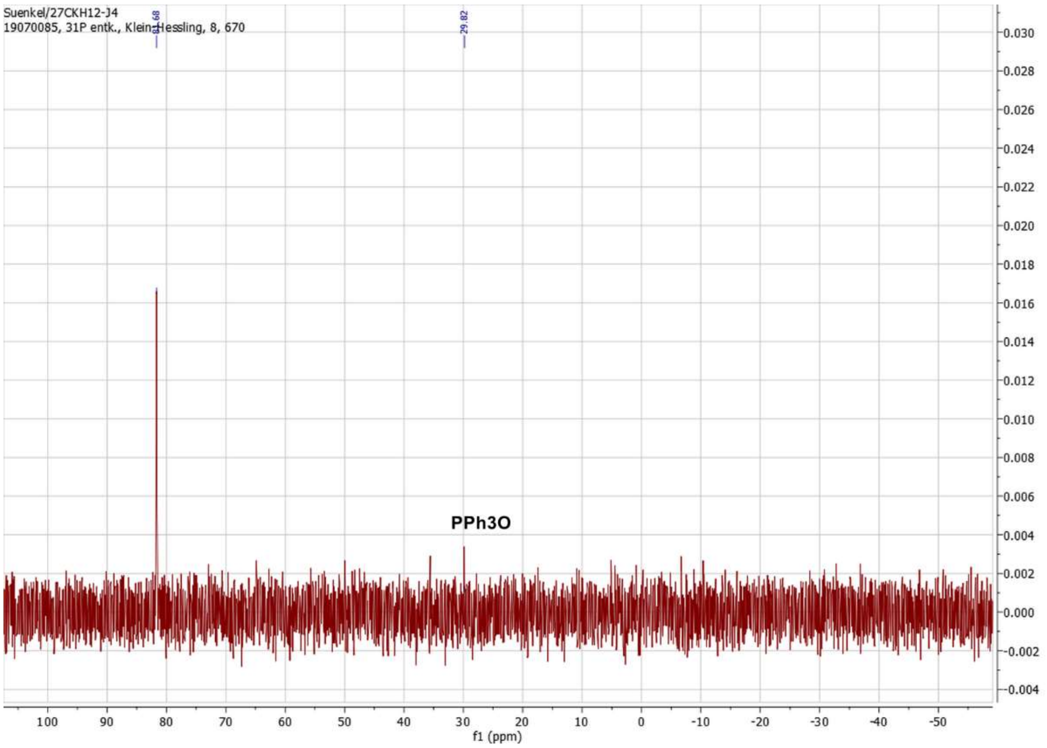

Figure S 25 $^{31}P\{^1H\}$-NMR spectrum (162 MHz, $CDCl_3$) of **11**

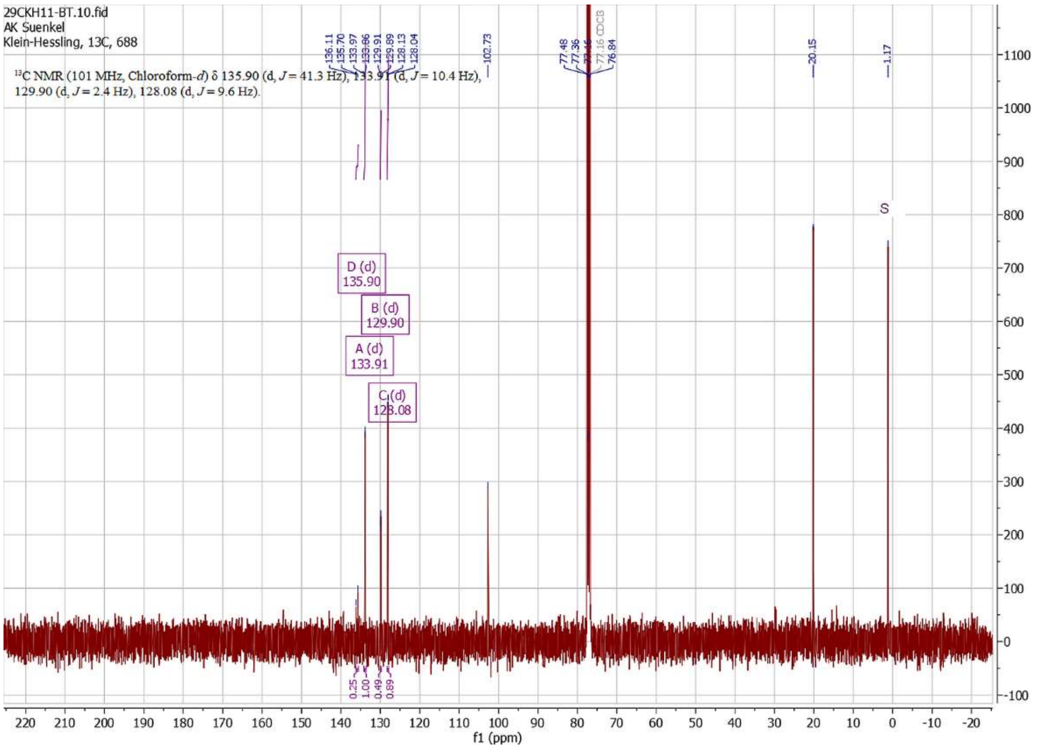

Figure S 26 $^{13}C\{^1H\}$-NMR spectrum (101 MHz, $CDCl_3$) of **11**

2. Mass spectra

General remark: mass peaks are marked according to: A = M^+, B = M^+ -2CO, C = M^+ -2CO – CH_3, C’ = M^+ - 2CO – 2 CH_3, D = M^+ - 2CO – PPh_3, E = M^+ -2CO – PPh_3 – CH_3

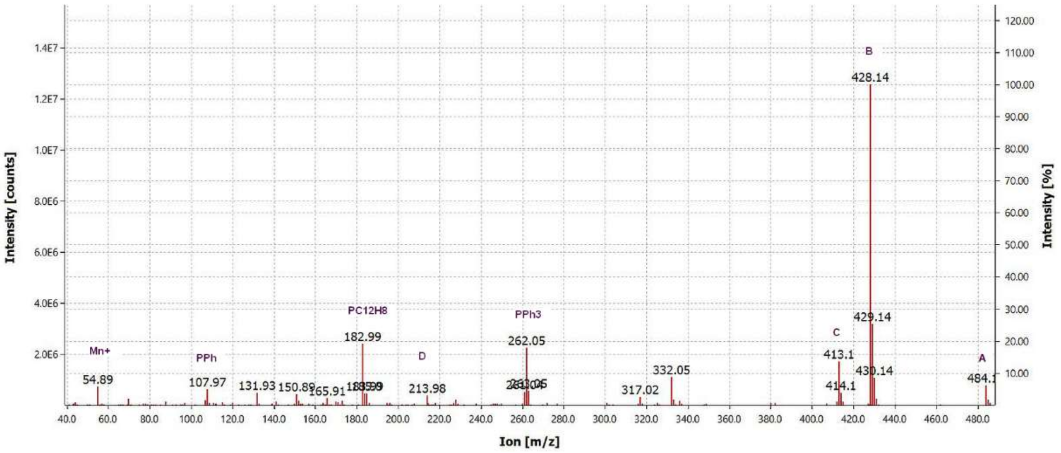

Figure S 27 DEI-MS of compound **1b**

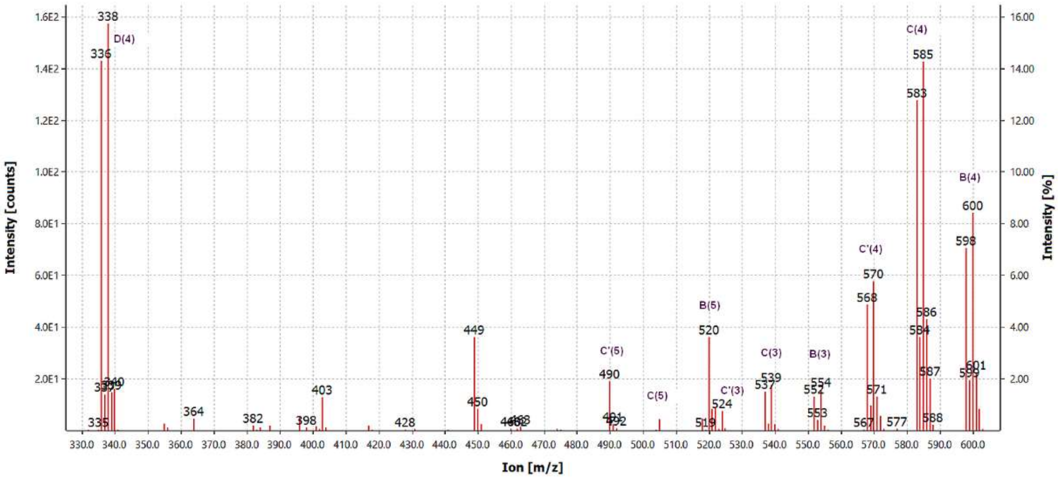

Figure S 28 DEI-MS of crude product from the reaction of **3** with 2.5 eq. LiTMP and Me_2S_2

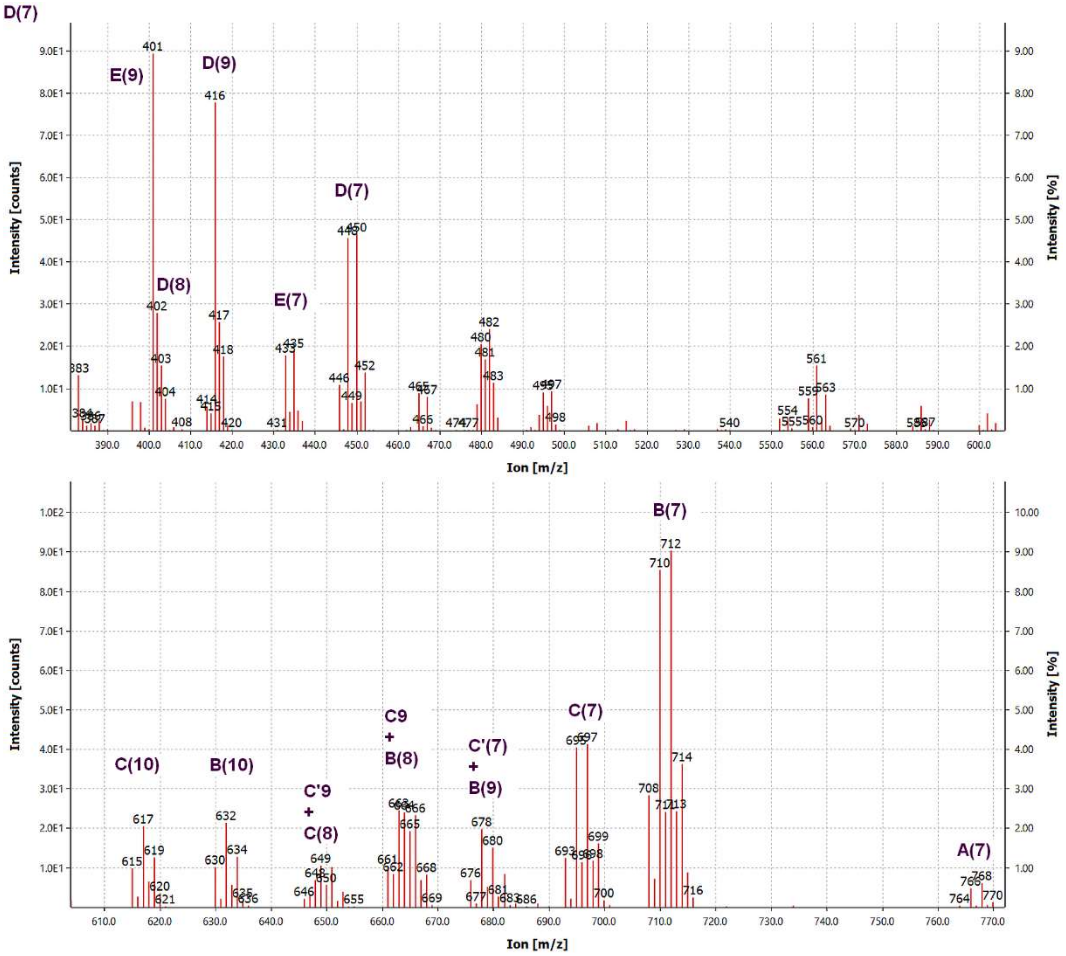

Figure S 29 DEI MS of the crude reaction mixture obtained from the reaction of **6** with 2.5 eq. n-BuLi/S_2Me_2 in THF at -78°C

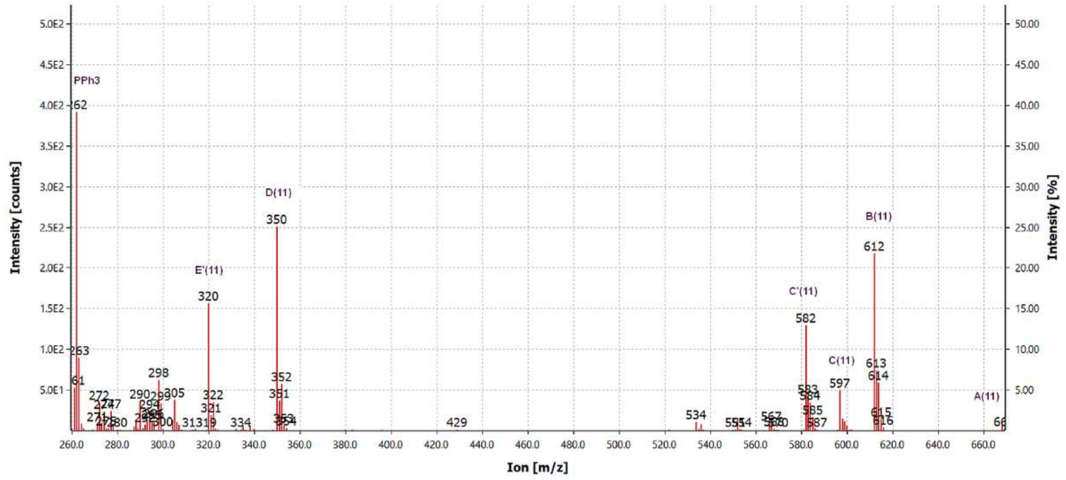

Figure S 30 DEI mass spectrum of **11**

3. Crystal and Molecular Structures

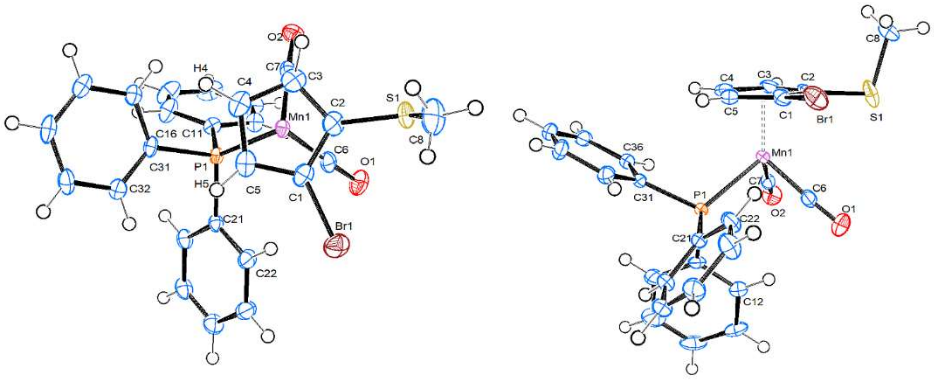

Figure S 31: Top and side view of the molecular structure of compound **2** (only one enantiomer shown; cyclohexane solvent not shown). Displacement ellipsoids at 50% probability.
